# Mechanism of Action of Non-Synonymous Single Nucleotide Variations Associated with *α*-Carbonic Anhydrase II Deficiency

**DOI:** 10.3390/molecules24213987

**Published:** 2019-11-04

**Authors:** Taremekedzwa Allan Sanyanga, Bilal Nizami, Özlem Tastan Bishop

**Affiliations:** 1 Research Unit in Bioinformatics (RUBi), Department of Biochemistry and Microbiology, Rhodes University, Grahamstown 6140, South Africa; asanyanga@gmail.com (T.A.S.); nizamibilal1064@gmail.com (B.N.); 2 Institute of Materials and Environmental Chemistry, Research Centre for Natural Sciences of the Hungarian Academy of Sciences, Magyar tudósok körútja 2, 1117 Budapest, Hungary

**Keywords:** precision medicine, carbonic anhydrase II, single nucleotide variation, allosteric effect, dynamic residue network analysis, MD-TASK

## Abstract

Human carbonic anhydrase II (CA-II) is a Zinc (Zn${}^{2+}$) metalloenzyme responsible for maintenance of acid-base balance within the body through the reversible hydration of CO${}_{2}$ to produce protons (H${}^{+}$) and bicarbonate (BCT). Due to its importance, alterations to the amino acid sequence of the protein as a result of single nucleotide variations (nsSNVs) have detrimental effects on homeostasis. Six pathogenic CA-II nsSNVs, K18E, K18Q, H107Y, P236H, P236R and N252D were identified, and variant protein models calculated using homology modeling. The effect of each nsSNV was analyzed using motif analysis, molecular dynamics (MD) simulations, principal component (PCA) and dynamic residue network (DRN) analysis. Motif analysis identified 11 functionally important motifs in CA-II. RMSD data indicated subtle SNV effects, while PCA analysis revealed that the presence of BCT results in greater conformational sampling and free energy in proteins. DRN analysis showed variant allosteric effects, and the average *betweenness centrality (BC)* calculations identified Glu117 as the most important residue for communication in CA-II. The presence of BCT was associated with a reduction to Glu117 usage in all variants, suggesting implications for Zn${}^{2+}$ dissociation from the CA-II active site. In addition, reductions to Glu117 usage are associated with increases in the usage of the primary and secondary Zn${}^{2+}$ ligands; His94, His96, His119 and Asn243 highlighting potential compensatory mechanisms to maintain Zn${}^{2+}$ within the active site. Compared to traditional MD simulation investigation, DRN analysis provided greater insights into SNV mechanism of action, indicating its importance for the study of missense mutation effects in proteins and, in broader terms, precision medicine related research.

## 1. Introduction

Carbonic anhydrases (CAs) are metalloenzymes responsible for the catalysis of the reversible interconversion of carbon dioxide (CO${}_{2}$) and water (H${}_{2}$O) to bicarbonate (HCO${}_{3}^{-}$ or BCT) and protons (H${}^{+}$), and the reaction is illustrated in Equation (1) [1,2]. At least six distinct CA families have been identified to date, namely, α (alpha), β (beta), γ (gamma), δ (delta), η (eta) and ζ (zeta) [3,4,5,6]. Vertebrates contain only the α family. The β-CAs are found in all organisms except for chordates [3,4,6]; γ-CAs are found widely in bacteria and plants in addition to methanogenic archaea [3,4]; δ-CAs are found in diatoms and in some marine algae [7]; and ζ-CAs are not found in some bacteria but only in diatoms [8]. It should be noted that the α family can also be found in bacteria. Although CAs are found in a variety of organisms, these enzyme families do not contain significant amino acid sequence similarity and are regarded as an example of convergent evolution [9,10].

(1) $$CO_{2}+H_{2}O⇌HCO_{3}^{-}+H^{+}$$

The α-CA proteins in humans are divided into four subgroups depending on intracellular location, cytosolic, mitochondrial, secreted and membrane associated, with each consisting of several isoforms. The isoforms across all subgroups total 15 enzymes. CA-I, II, III, VII and XIII belong to the cytosolic subgroup, while CA-IV, IX, XII and XIV are members of the membrane associated subgroup. CA-VA and VB are mitochondrial enzymes, and CA-VI is part of the secreted subgroup [1,10,11]. CA-VIII, X and XI are regarded as the carbonic anhydrase related proteins (CARP) and do not possess CO${}_{2}$ hydration activity. From the α-CAs, CA-II has the highest rate of reaction and assists in the maintenance of homoeostasis within the body [12]. Reaction substrates and products (CO${}_{2}$, HCO${}_{3}$${}^{-}$ and H${}^{+}$) are essential for the regulation of biological processes such as, but not limited to, respiration, cerebrospinal fluid (CSF) formation and bone resorption within cells [13,14]. CA-II consists of 260 residues and a Zinc ion (Zn${}^{2+}$) within the active site that is in tetrahedral coordination geometry with three histidine residues (His94, His96, His119) and a water molecule [1,6,10,11,15,16]. These coordinating residues are also known as coordination ligands. The active site also comprises of hydrogen bonded waters that form part of the proton transfer network with His64 acting as the main proton shuttle, transporting protons to and away from the active site [17]. His64 facilitates its proton shuttling role by alternating between two conformations (“in” and “out”) during catalysis [16,17,18,19] or through tautomerisation of the histidine ring [20].

Three CO${}_{2}$ binding pockets have been identified in CA-II and are located approximately 3–4, 5–7 and 10–12 Å away from the Zn${}^{2+}$ [21,22,23,24]. For the purposes of this study, these pockets have been designated as the primary, secondary and tertiary binding pockets, respectively. The primary binding pocket is made up of hydrophobic residues Val121, Val142, Leu197 and Trp208, while the secondary CO${}_{2}$ binding site is made up of aromatic residues Phe66, Phe95, Trp97 and Phe225. The tertiary binding pocket comprises the residues Trp7, His64, Thr199, Pro200 and Asn243 and is located along a tunnel leading to the primary pocket. Of the three binding pockets, the secondary pocket is the only non-catalytic binding pocket, and its role in CA-II is yet to be fully investigated [1,21,22,23,24].

To assist with protein stability, CA-II contains two groups of aromatic residues known as the primary and secondary aromatic clusters. The primary aromatic cluster consists of the residues Trp5, Tyr7, Trp16 and Phe20, and joins the N terminal to the rest of the protein [1]. The secondary aromatic cluster is larger and is comprised of the residues Phe66, Phe70, Phe93, Phe95, Trp97, Phe175, Phe178 and Phe225 [1,25,26].

In the absence of CA-II, CO${}_{2}$ is hydrated at a rate constant between 0.030 and 0.15 s${}^{-1}$, compared to 1 × 10${}^{6}$ s${}^{-1}$ in the enzyme mediated reaction [15,27,28,29,30]. The large difference in reaction rates coupled with the importance of CA-II to other biological processes indicates that any impairment to the function of CA-II could have detrimental effects to cells and the body. In humans, poor CA-II function causes CA deficiencies resulting in the phenotypes’ osteopetrosis with renal tubular acidosis and cerebral calcification [31]. Advances in genomic research identified non-synonymous single nucleotide variations (nsSNVs) occurring in CA-II to be the main cause of these diseases [32,33]. Numerous studies have been done associating CA-II SNVs with CA deficiencies. For instance, research in 2004 by Shah et al. [34] identified 11 novel CA-II mutations, such as G144R, in individuals suffering from CA deficiencies leading to osteopetrosis with renal tubular acidosis and cerebral calcification. The changes to the amino acid sequence of CA might influence residue interactions and communication within the protein resulting in poor enzyme function and stability causing protein deficiencies. As variations might lead to dysfunctional proteins and cause the indicated diseases, it is important to understand the mechanism of these SNVs to identify “activator” compounds reversing the effect of variations and rescuing the protein function.

To date, most of the research into CA has focused on inhibition for the management of conditions such as, but not limited to, glaucoma and altitude sickness, which are related to the overexpression of CA-II [35,36,37,38]. CA inhibitors have also found use as diuretics [38,39]. However, prolonged use of CA inhibitors is not without consequence; for example, prolonged use of acetazolamide is associated with a reduction in osteoclast function and bone resorption [40] that could potentially lead to osteopetrosis. Factoring in the potential presence of SNVs and their effect on CA-II function, specific inhibitors would have varying efficacies across different individuals depending on the SNV that is present within the CA-II proteins, highlighting a research gap for precision medicine related studies for CA inhibitors.

The aim of the current study is to characterize the structural and functional effects of six validated nsSNVs (K18E, K18Q, H107Y, P236H, P236R and N252D) on CA-II protein structure as proposed previously [41,42], by combining homology modelling, molecular dynamics (MD) simulation, principal component analysis (PCA) and dynamic residue network (DRN) analysis [41,42] to identify underlying mechanisms responsible for CA-II deficiencies. Previous studies have focused only on MD simulations to analyze the effect of SNVs in CA-II. Within this research, not only have we used MD to analyze the variant effects but we have also employed DRN to analyze SNV effects on residue and protein network communication. DRN analysis demonstrated differences to the variant mechanisms of action, and revealed all six SNVs to be associated with allosteric effects in variant proteins. DRN, further, showed that Glu117 is the most important residue within the protein. We were also able to predict that H107Y is the most deleterious variant due to its direct reduction to Glu117 communication in all three protein states (apo, BCT and CO${}_{2}$ bound). In general, this research enhances the importance of the previously proposed approach in variant analysis [41,42], in that MD alone is insufficient to understand the effects of missense mutations to protein structure, and shows the importance of coupling MD with DRN analysis. This method is particularly important for precision medicine related studies, which aim to analyze the cause of disease at the molecular level and then to utilize targeted treatment for patients.

## 2. Results and Discussion

In this study, six validated and pathogenic SNVs of CA-II were investigated using a combination of motif analysis, MD, PCA and DRN analysis to determine potential variant effects on the protein structure and function at the molecular level.

### 2.1. Six Disease Related SNVs and Their Spatial Positions Are Identified

Data retrieval from the Ensembl [43] and HUMA [44] databases identified six validated SNVs with pathogenic effects (Table 1). Even though there were several more SNVs, we limited our CA-II dataset to those validated within dbSNP via frequency, or via cluster, and containing a phenotype annotation. Table 1 also presents the predicted variant effects on protein structure using the Variant Analysis Portal (VAPOR) [44]. VAPOR is a SNP/SNV analysis tool that combines PhD-SNP [45], PROVEAN [46], FATHMM [47] and PolyPhen-2 [48] to predict SNV presence as either damaging or tolerated. VAPOR also incorporates I-Mutant 2.0 [49] and MUpro [50] to predict SNV effects on stability [44]. MUpro analysis showed that all SNVs are expected to cause reductions to protein stability. Data in Table 1 also presents the global minimum allele frequency (MAF) of the SNVs obtained from gnomAD [51]. Data indicates that N252D occurs at the highest frequency.

Homology modeling of variant proteins was performed by Schrödinger Prime [52,53]. Z-DOPE (normalized discrete protein energy) score [54] and Ramachandran plot were used to assess protein model quality and select the best structure for each case. The z-DOPE statistical method shows that all structures have scores less than −2.00 (Table 1). This indicates that models resemble native protein structures. Within the scope of the studied literature, no evidence as to the possibility of variant linkage disequilibrium in CA-II was observed. As a result, all modeled proteins contained one SNV only, and no structures with combinations of SNVs were generated and analyzed.

**Figure 1:**
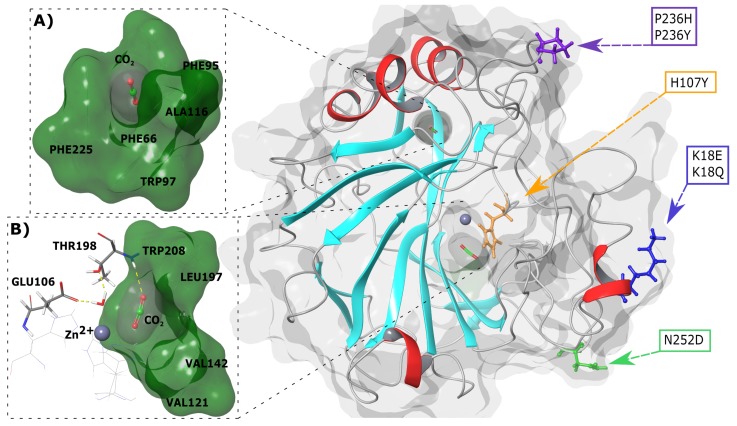
3D structure of human CA-II WT protein and SNV locations. (**A**) Secondary CO${}_{2}$ binding pocket. (**B**) Primary CO${}_{2}$ binding pocket. Cyan and red secondary structure represents beta sheets and helices, respectively. The grey sphere represents the Zn${}^{2+}$. Representations were generated using Schrödinger Maestro and Inkscape.

Next, 3-dimensional (3D) spatial analysis of variant location in relation to the active site and CO${}_{2}$ binding pockets was done. Figure 1 presents the SNV positions within the CA-II protein. K18E and K18Q are located within a helix whereas the other variants are present in loop regions. H107Y is located within the protein interior and closest to the active site and primary CO${}_{2}$ binding pocket, whereas the other variants are positioned towards the exterior of CA-II. The primary and secondary binding sites of CO${}_{2}$ are also presented in Figure 1A,B. As the secondary CO${}_{2}$ binding pocket is acatalytic, during catalysis (see Equation (1)) the substrates BCT and CO${}_{2}$ both bind to the primary pocket.

### 2.2. Identified SNVs May Have an Indirect Effect on Protein Structure and Function

To date, numerous researches have been conducted on CA-II structure. These studies allowed the identification of key residues essential to CA-II structure and function as presented in Table S1. Interestingly, it is evident that none of the selected variants are located at important residues, suggesting that as opposed to having a direct effect on the protein structure, variant action may occur via indirect or secondary mechanisms. This hypothesis has been uncovered in the rest of the article.

### 2.3. Identified SNVs Are Located within or around the Highly Conserved Motifs

Important functional residue groups in protein families are generally highly conserved. Hence, as a next step, motif analysis was performed across all selected α-CA proteins (Table S2) to identify conserved motifs that might contain new functional residue groups in addition to those in Table S1. The default length of the motifs that can be searched in the Multiple Expectation Maximization for Motif Elicitation (MEME) program [55] is up to 50 residues. Short linear motifs to that function in protein–protein interactions are known to be between 3 and 11 residues [56,57]. To our knowledge, there is no defined length to the functional motifs within a protein; hence, we set MEME parameters to lengths of 3–20 amino acids to include possible motifs marginally longer than average short linear motifs as applied in our previous study [56]. MEME calculations initially gave 100 motifs across all α-CA sequences. Motif pairwise correlation data analysis reduced the final dataset to 77 as explained in the Materials and Methods (Section 3.2).

**Figure 2:**
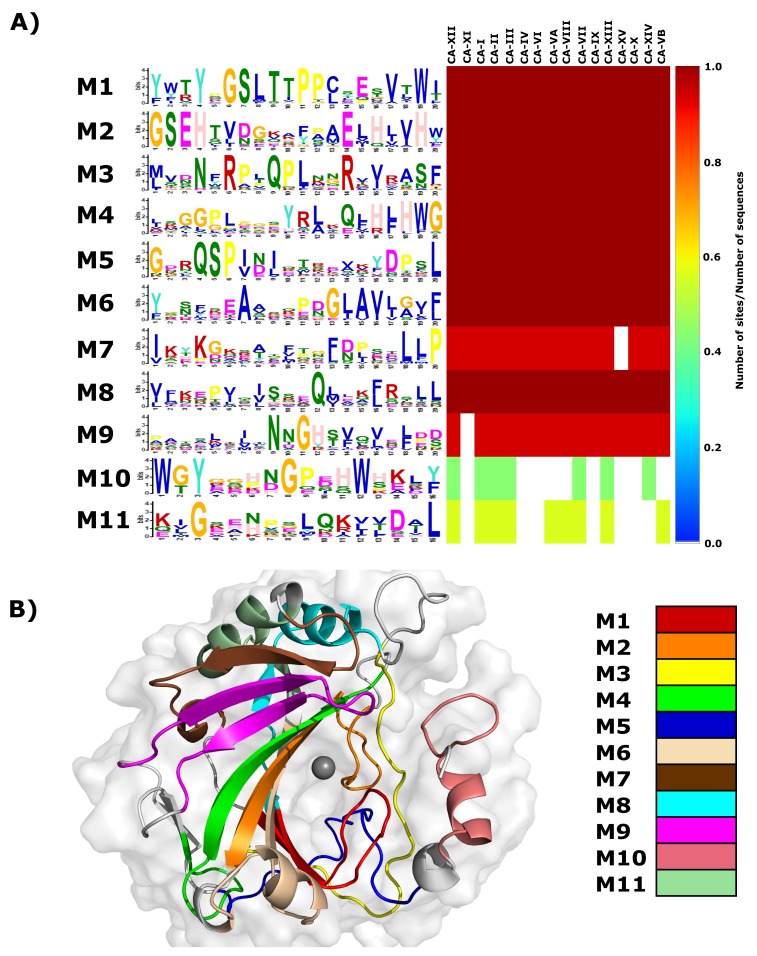
Motifs present in the α-CA family. (**A**) Web logo presenting residue conservation within a motif in each sequence and a heat map showing motif conservation as the number of motif sites per total number of protein sequences. A value of zero is for motifs that are not conserved in any sequence, whereas a value of 1 shows that motif exists in all selected sequences. The prefix ’M’ represents the word “motif”. (**B**) Motif location in 3D space within CA-II. The grey sphere represents the Zn${}^{2+}$. Representations were generated using Schrödinger Maestro and Inkscape.

A heat map of the remaining 77 motif dataset is presented in Figure S1, with motif conservation represented as the number of sites per total number of protein sequences. Motifs are numbered according to the MEME output. From the heat map data, it is evident that only motifs 1–11 are conserved in CA-II and have significant E-values (Table 2). As a result, motifs 1–11 were selected for further analysis. Data in Figure 2A presents a combination of the motif logo and conservation results. The motif logo is a representation of the amino acids present in each motif (M1–M11), and shows the conservation of each protein residue in that motif sequence. The heat map highlights motif conservation within the selected proteins (Table S2). A value of 1 represents 100% motif conservation in all sequences, whereas a value of 0 suggests that the motif does not exist in any of the proteins within the selected set. Since the motif analysis included the non-catalytic CA isoforms CA-VIII, CA-X and CA-XI, it should be noted that the most significantly discovered motifs are most probably important for the maintenance of structure and stability.

Heat map results in Figure 2A demonstrate that motifs 1–6 and 8 are conserved across all α-CA proteins, indicating that residues within these motifs have most likely important function and/or structural (i.e., stability) roles within the α-CAs. Motif 7 is conserved in all except CA-XV, and motif 9 is conserved in all except CA-XI. Motif 10 is conserved in all cytosolic CAs (I, II, III, VII and XII) and is also conserved in two membrane CA proteins (XII and XIV). CA-XII and XIV are single pass type I membrane proteins with the N-terminus existing extracellular and C-terminus towards the cytosol [11]. Existence of these proteins within the cytosol suggests that this motif could play a role in protein function, solubility and/or stability within the cell. Motif 11 is conserved in all α-CAs located within the cytoplasm, and includes acatalytic isoform CA-VIII. Conservation within CA-VIII suggests that this motif most likely is important for protein stability within the intracellular environment, as opposed to catalytic function. These motifs are mapped to the 3D structure of CA-II (Figure 2B).

As a next step, we checked to see if motifs contain Table S1 residues as well as variant data. Where applicable, the mapping of residues in Table S1 onto motifs allowed for the assignment of motif function. Since proteins are networks of residues communicating with one another [41,42], the function of specific protein residues is affected by other surrounding residues. Assignment of function via residue mapping was conducted as follows, using motif 1 as an example. Data in Table 2 shows motif residue range, sequence and associated E-value, and from the table, it is noted that motif 1 contains the residues 190 to 209. Within this group of residues exists, Thr198 and Thr199 that are essential in the catalytic pathway via the catalytic orientation of the Zn${}^{2+}$ water ligand molecule and active site water network coordination (see Table S1). In addition to Thr198 and Thr199, motif 1 also contains Leu197, Pro200 and Trp208 that are essential for primary and tertiary CO${}_{2}$ binding pocket formation. Therefore, a combination of the functions Leu197, Thr198, Thr199, Pro200 and Trp208 in CA-II suggests that motif 1 is involved with catalysis and CO${}_{2}$ binding pocket formation. A similar process was used to assign functions of the remaining 10 CA-II motifs. Assigned motif functions are presented in Table 2.

Results in Table 2 further indicate the respective SNV positions in each motif. K18E and K18Q are present on motif 10 while H107Y is located on motif 2. The variants P236H and P236R are not located on any conserved motifs, whereas N252D is present on motif 3. Analysis of SNV locations with respect to their motifs suggests that H107Y could have the greatest effect on protein structure and function. P236H and P236R are located between the two highly conserved motif 3 and motif 8. Variant presence could indirectly influence these motifs. In addition, as none of the variants are located on motifs responsible for catalytic mechanism, SNVs could still influence catalysis due to stability decreases in or near active site residues. Potential effects of variants on enzyme mechanism were also investigated later within this article.

### 2.4. Zn${}^{2+}$ Parametrization Helps the Ion to Maintain Its Position over MD Simulations

As traditional MD force fields are designed for protein amino acids, these built-in force fields are incapable of handling metals and potential interactions with amino acids. A consequence of this would be metal escape from the active site of the protein during the MD simulations and diminishing their accuracy. Within this study Zn${}^{2+}$ force field parametrization was essential to govern how Zn${}^{2+}$ interacts with its coordinating atoms and prevent it from escaping the active site. Parametrization was performed using AmberTools17 [58]. Gaussian 09 [59] quantum mechanical (QM) optimizations showed no evidence of bond breakage between Zn${}^{2+}$ and coordinating atoms, giving a strong indication that the derived force field parameters would hold the Zn${}^{2+}$ in place during MD. In addition, bond length measurements in between the Zn${}^{2+}$ and coordinating atoms were compared to those previously derived by Harding [60], and by Bernadat et al. [61]. Results indicated that bond lengths calculated by the Metal Centre Parameter Builder (MCPB) [62] fell within previously reported ranges of His < 2.03 Å and H${}_{2}$O < 2.18 Å [60,63]. Bond angles calculated within this study were also similar to those derived in previous research [61]. Parametrization of the active site further determined the Zn${}^{2+}$, His94 (ND), His96 (ND) and His119 (NE) to have atom charges of 0.592, −0.089, −0.033 and −0.160, respectively. Results agree with those previously calculated by Bernadat et al. [61] that show Zn${}^{2+}$ to have a charge less than one, and the coordinating His ND and NE atoms having lower negative charge compared to their standard charges [61]. The calculated final Zn${}^{2+}$ parameters are presented in Table S3.

### 2.5. Global Level Analyses Hint at the Functional/Structural Effect of Certain Variants

21 protein structures (WT and six variant proteins for each state; apo, BCT and CO${}_{2}$ bound) were taken into 200 ns MD simulations. The trajectories were analyzed using root mean square deviation (RMSD), PCA and radius of gyration (Rg) metrics to observe potential variant structural effects, in the absence and presence of BCT and CO${}_{2}$ molecules at the global level.

#### 2.5.1. Proteins with Variations Occupy Different Conformational Spaces to the WT

#### RMSD Analysis

**Figure 3:**
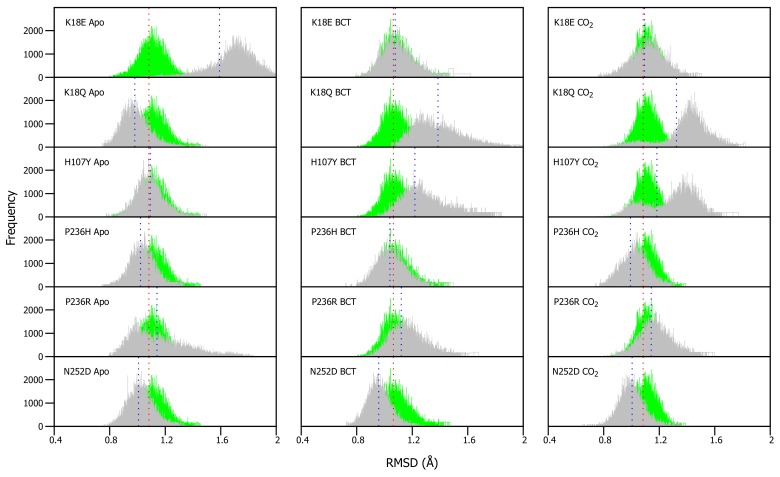
α-carbon RMSD distribution of the WT and variant proteins over the 200 ns MD simulation. The green and grey histograms indicate the RMSD distribution of the WT and variant protein, respectively. The red and blue dashed lines represent the mean RMSD of the WT and variant proteins, respectively. Variant apo, BCT and CO${}_{2}$ proteins are each plotted against the WT apo, BCT and CO${}_{2}$ bound proteins, respectively. The x-axis represents RMSD of sampled conformations during MD simulation, whereas the *y*-axis (Frequency) represents number of times a specific conformation was sampled.

As VAPOR results (Table 1) predicted a decrease in stability within the variants, RMSD differences between WT and variant proteins were expected. RMSDs of WT and variants were calculated for each frame over the MD simulation for each protein system and compared (Figure S2). Overall RMSD values did not show any drastic changes in the presence of variations, probably an indication of more subtle effect. To be able to understand the variation effect and to observe potential discrete conformational changes in proteins, RMSD distribution histograms of the WT and variant proteins were generated, as previously applied in Penkler and Tastan Bishop 2019 [64] (Figure 3). WT and variant histogram width is the number of conformations sampled by proteins over the MD simulation. Frequency represents number of times a specific conformation was sampled during the MD simulation, and the histogram peaks are indicative of the most commonly occupied conformation. Variant apo, BCT and CO${}_{2}$ proteins were plotted against the WT apo, BCT and CO${}_{2}$ proteins, respectively. The variant apo structures were compared to the WT apo protein, whereas the variant BCT proteins were compared to the BCT bound WT protein, and variant CO${}_{2}$ proteins were compared to the WT CO${}_{2}$ bound protein.

Figure 3 presents the average RMSD differences between the WT and variant proteins. Though the differences are small, previous research in 2004 by Almstedt [32] proved that residue movements and rotations as small as 0.3–0.4 Å are enough to destabilize CA-II. To check the significance of our results, statistical calculations were performed (Table S4). Using a 5% level of significance, statistical data indicated that the changes and differences to structural conformations are statistically significant between the respective WT and variant proteins.

Apo protein structure analysis (Figure 3) shows that the H107Y has the closest average structure to that of the WT, whereas K18E has the largest structural differences from the WT. In addition, with exception to K18E and P236R, it is observed that the average RMSD coincides with the histogram peaks. This finding can be explained by the plot shape of K18E and P236R. The wide histogram base suggests that these variants sampled the most conformations during MD. Statistical results however present an interesting finding with regards to the WT CO${}_{2}$ bound protein and H107Y apo variant. These two proteins are not statistically different (*p*-value: 0.5124) indicating some conformational similarity. In the presence of substrate BCT, the P236H has the closest average RMSD to that of the WT, while K18Q has the largest average RMSD difference to that of the WT.

When the substrate CO${}_{2}$ is bound, P236R has the closest average RMSD to that of the WT, whereas, K18Q has the largest difference in average RMSD from the WT protein. As was observed when BCT is bound, variants K18Q and H107Y are associated with the greatest conformational changes. K18Q and H107Y histograms also demonstrate two RMSD peaks each, indicating that during MD, K18Q and H107Y occupied two conformations (one is more dominant than the other). Using the WT as reference, the conformations which are represented with the peaks closest to the WT, at RMSD of 1.1 Å would most likely be the ones capable of catalysis by comparison of structures sampled during MD simulation for WT and variant proteins.

#### PCA Analysis

RMSD data represents a 2D interpretation of the conformations sampled by the proteins during MD. PCA analysis was performed to expand on the RMSD data and to observe the 3D conformational sampling and internal dynamics of the WT and variant proteins. Figure S3 shows the 3D PCA plots of the WT and variant proteins. The eigenvalue fraction of each PC is presented in Table S5 and indicates that most of the conformational sampling space is covered by PC1 and PC2. PC1 represents the largest possible variance while PC2 is representative of the second largest variance within the structures. Conformations associated with low free energy are expected to be more stable.

In the apo form, the WT samples two distinct protein conformations along PC1 and one conformation along PC2. Though two conformations are sampled along PC1, RMSD results only show evidence of one major conformation in Figure 3. This can be explained by size of the low energy well in Figure S3. The larger well suggests that the majority of the structures are at that conformation, and therefore these conformations represent the peak observed in the RMSD results. When BCT is bound, the protein samples a larger conformation space than that of the apo and CO${}_{2}$ bound structures. The BCT bound conformations also have a higher free energy. When CO${}_{2}$ is bound, the WT forms two structures along PC1; however, only one structural cluster has low energy, suggesting that this is the conformation observed within RMSD results.

K18E and K18Q data indicate that although the variants occur at the same position, the mechanism of action could be different. When BCT is bound, K18E occupies a larger conformational sampling space than that of K18Q. Free energy data suggests that the BCT bound structures in K18E have higher energy than those of K18Q. When CO${}_{2}$ is bound to K18Q, two conformational clusters are formed along PC1, and these conformations are also observed within the RMSD results. When BCT is bound to H107Y, compared to the WT, the variant has higher free energy and samples more conformations. This indicates evidence of variant associated greater conformational changes and supports findings in RMSD. When substrate CO${}_{2}$ is bound to H107Y, the results indicate two distinct low energy conformational clusters that are also observed within the RMSD results. P236H and N252D conformations exhibit the highest free energy when BCT is bound, compared to the WT and other variant proteins. The apo protein of P236R demonstrates evidence of potential instability. The variant forms two conformational clusters along both PC1 and PC2. Occupation of multiple conformations is indicative of variant associated conformational changes, and this finding agrees with that observed within the RMSD results.

#### Rg Analysis

The RMSD and PCA analyses of the WT and variant proteins allowed for the determination of SNV effects on protein; conformation, stability and possible functional consequences in global level. To expand on these results, the Rg of all protein systems were calculated. Calculation of Rg allows for identification of potential relationships between protein compactness, conformation and stability within the variant proteins [65]. Considering the effects of protein compactness on enzymatic function, decreases would result in key enzyme residues moving away from each other, whereas increases in protein compactness would result in residues moving closer to each other. Previous CA-II studies have suggested that residue-residue distance has an effect on enzyme kinetics, and protein residues can be neither too close nor too far from each other for optimal enzyme activity [16,27,66,67,68,69].

**Figure 4:**
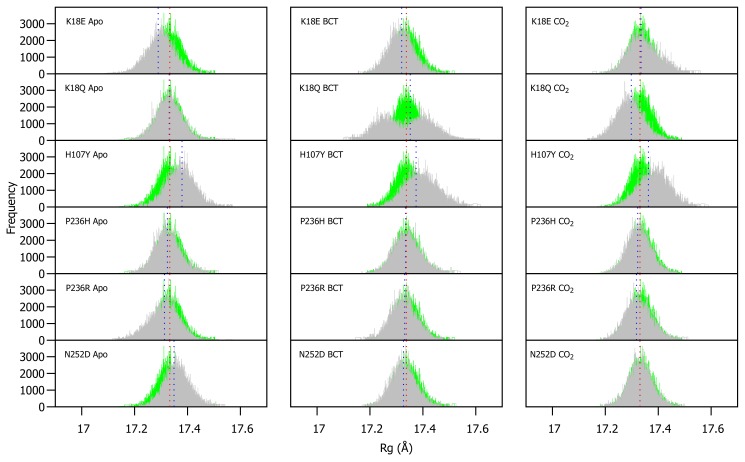
Rg distribution of the WT and variant proteins over the 200 ns MD simulation. The green and grey histograms indicate the Rg distribution of the WT and variant protein, respectively. The red and blue dashed lines represent the mean Rg of the WT and variant proteins, respectively. Variant apo, BCT and CO${}_{2}$ proteins are each plotted against the WT apo, BCT and CO${}_{2}$ bound proteins, respectively. The x-axis represents Rg of sampled conformations during MD simulation, whereas the *y*-axis (Frequency) represents number of times a specific conformation was sampled.

WT and variant Rg were calculated for each frame over the MD simulation for each protein system and compared (Figure S4). As observed in RMSD, variant presence shows minimal effect on protein compactness. Figure 4 presents a histogram of the Rg distribution between the WT and variant proteins. As with the RMSD distribution, variant apo structures are compared to the WT apo protein, whereas the variant BCT proteins are compared to the BCT bound WT protein, and variant CO${}_{2}$ proteins are compared to the WT CO${}_{2}$ bound protein.

The Rg distribution in Figure 4 indicates that H107Y is associated with a reduction to compactness for all three protein states. When the H107Y has substrates BCT and CO${}_{2}$ bound, histograms have a wider base compared to the WT. This observation can be explained by the previous RMSD findings whereby the proteins exhibited greater conformational sampling. When BCT is bound to K18Q, an interesting result is observed. The wide base of the Rg distribution histogram demonstrates evidence of greater Rg sampling compared to the WT. In addition, the histogram exhibits two peaks, indicating major changes to compactness during MD. Comparison of RMSD and Rg distribution peaks for K18Q suggests that the conformations sampled during MD (RMSD) could cause a shift to the protein centre of mass and cause the two observed Rg peaks. When CO${}_{2}$ is bound to K18Q, an increase in protein compactness is observed.

Within this section we analyzed potential global functional and/or structural effects associated with SNV presence and noted that the variants have some subtle effects where the significance is supported by statistical calculations on CA-II compactness and conformation (see Figure S4). At the 5% level of significance, statistical data shows that WT and variant protein compactness is significantly different for most of the protein systems. The WT apo and the CO${}_{2}$ bound K18E variant do however share similar compactness (*p*-value: 0.06527). The CO${}_{2}$ containing N252D and BCT bound P236R variants indicate no significant differences to protein compactness when compared to the CO${}_{2}$ bound WT protein (*p*-values: 0.1479 and 0.3288, respectively). In the following section we take this research forward to identify residues that are responsible for the global effects observed.

### 2.6. Residue Level Analysis Reveals Further Differences between WT and Variant Protein Systems

In the first part of residue level analysis, we looked at the root mean square fluctuation (RMSF) values. WT and variant protein system RMSF values are presented in Figure S5. To improve data resolution, ΔRMSF between the WT and variant proteins was calculated for apo, BCT and CO${}_{2}$ protein states by subtracting WT RMSF from the variant RMSF data. Thus, a positive ΔRMSF is indicative of higher flexibility in variant residues, and a negative ΔRMSF shows a reduction in RMSF within the variant residues.

**Figure 5:**
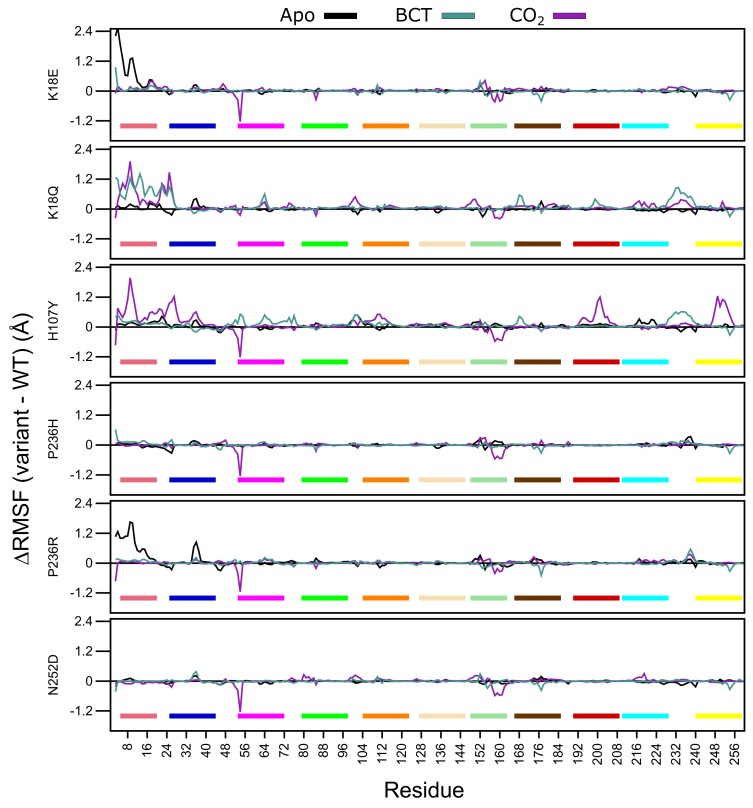
Δα-carbon RMSF comparison of the WT and variant proteins (variant minus WT). Respective motifs are indicated as bars at the bottom of each plot. Motif colors correspond to those in Figure 2B.

Analysis of Figure 5 ΔRMSF data demonstrates that for the apo proteins, SNV presence only influences the first 20 N-terminus residues of K18E and P236R. This group of residues are members of motif 10 (Table 2), and contain residues (Trp5, Tyr7, Trp16 and Phe20) within the initial aromatic cluster that are involved in maintaining CA-II stability. Increases to flexibility of residues in this region might have detrimental effects on protein stability. The previously observed RMSD results for K18E and P236R could be due to the increases in the fluctuations in motif 10 residues. Comparing the RMSF values between the two variant proteins, data indicate that the first 20 residues of K18E are more flexible than those of P236R.

When BCT is bound to the variant proteins, SNV effects are observed to the greatest extent in K18Q and H107Y. Both proteins are associated with increases to RMSF between residues 230 and 240. These groups of residues are located between motif 3 and motif 8; hence, increases to the flexibility of these residues could have an impact on stability. K18Q shows increases to the flexibility of motif 10 residues containing Trp5, Tyr7, Trp16 and Phe20 of the initial aromatic cluster. This observed RMSF increase could explain the potential instability and greater conformational sampling noted within the RMSD results. The substitution of Lys with Gln at position 18 is also associated with increases to RMSF. With respect to H107Y, RMSF increases are observed in some residues located in motif 2 (104–124), motif 7 (166–186) and motif 9 (53–73). RMSF increases to these motifs highlight potential implications for H107Y function and stability. Motif 2, motif 7 and motif 9 are all involved in enzyme stability (Table 2). The increase in the flexibility of these residue groups could explain the potential instability and greater conformational sampling observed within the RMSD when BCT is bound. In addition, flexibility increases in these motifs also suggest potential implications on enzyme function, as both motifs include active site residues and could have implications on enzyme function. This could also explain the poor activity associated with this variant.

When CO${}_{2}$ is bound, all variants with the exception to K18Q exhibit large RMSF decreases to residues 53–54 and 157–162. Residues 53–54 are located on motif 9. However, as the decrease to RMSF is not spanning multiple residues, variant presence could have minimal effect on the function of motif 9. Comparison of RMSD and RMSF results of K18Q and H107Y with the other variants when CO${}_{2}$ is bound highlights the potential role of motif 11. Both K18Q and H107Y are the only variants exhibiting RMSF increases in motif 10 residues Trp5 and Tyr7 of the initial aromatic cluster; however, all variants show RMSF decreases between residues 157 and 162. Noting that only K18Q and H107Y exhibit potential instability and greater conformational sampling in RMSD, this could be associated only with motif 10. This finding suggests that motif 11 may not play a role in CA-II stability.

H107Y shows most changes to RMSF when CO${}_{2}$ is bound compared to the other variants. In addition to the flexibility increases in motif 10 residues, RMSF increases are also noted in residues 192–208 (motif 1) and 246–254 (motif 3). Overall flexibility increases demonstrate that SNV effects are centered around the active site of the protein, and RMSF increases to Thr199 and Pro200 show potential effect on the binding of CO${}_{2}$ to the tertiary pocket. This result suggests that, compared to the other variants, H107Y could have the greatest effect to enzyme function.

Throughout all variants, the presence of substrates BCT and CO${}_{2}$ has the greatest effect on RMSF. Overall data further supports the initial finding that the SNV presence has an allosteric effect on CA-II structure and function. This is evidenced by RMSF changes away from SNV positions. In the next sections, we explore DRN to analyze the SNVs effect at residue level and identify the potential allosteric effects of variants.

### 2.7. Short Range Effects of Each Variation Are Deciphered Using Weighted Contact Map Analysis

Since RMSD and Rg analysis showed subtle changes associated with SNV presence, network analysis was performed to obtain a more thorough and robust understanding of variant effects on the CA-II protein. Through previous research, network analyses has proved useful in the identification of key regions and residues essential for communication, function and stability in proteins [70,71,72]. In 2017, Brown et al. [41] utilized DRN to successfully investigate SNV effects on protein dynamics. Here, contact map analysis was performed to identify SNV associated short-range changes to the protein network and potential variant mechanisms. Contact maps show all weighted/frequency of interactions occurring between a network of residues within 6.7 Å over the MD simulation. These weighted contacts include all interactions such as, but not limited to, van der Waals, hydrogen bonds and/or electrostatic interactions [42].

**Figure 6:**
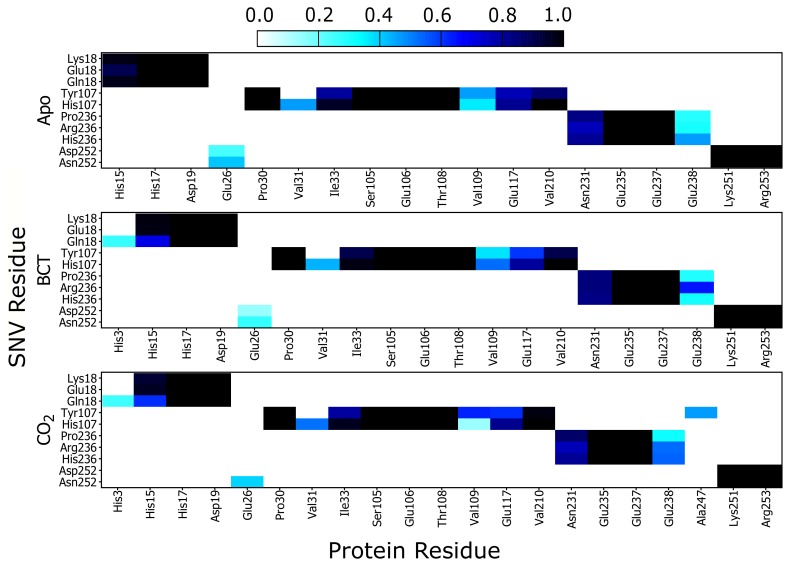
Heat map presenting the contact map weighted interactions between the respective SNV residues in; K18E, K18Q, H107Y, P236H, P236R and N252D with neighboring CA-II residues.

Results in Figure 6 show a heat map of the weighted contacts (frequency of interaction) occurring between all variant protein SNVs and their corresponding neighboring residues. Weighted contacts with the value 0 indicate that there is no contact occurring between the two residues, whereas a value of 1 shows that respective amino acids are constantly interacting over the MD simulation.

Analysis of K18E contact maps in Figure 6 indicates no changes to interactions occurring between either Lys18 or Glu18 and the other protein residues; however K18Q demonstrates that when BCT or CO${}_{2}$ are bound, Gln18 forms new interactions with His3. Since His3 is not conserved nor located on any CA-II motif group (Table 2) formation of these interactions may be of minimal importance in maintaining enzyme structure and function. On the other hand, comparison of His15 interaction analysis with Glu18 (K18E) and Gln18 (K18Q), with the corresponding RMSD results, shows an interesting finding. Decreases in interactions between His15 and Glu18/Gln18 are associated with potential instability and greater conformational sampling (Figure 3). Glu18 in the K18E apo protein shows a decrease to interactions with His15 (Figure 6), and the resulting RMSD histogram for K18E apo in Figure 3 exhibits potential instability and greater conformational sampling. Gln18 in K18Q indicates decreases to interactions with His15 only when substrates are bound, and the resulting RMSD distribution of K18Q indicates potential instability and greater conformational sampling when BCT and CO${}_{2}$ are bound. Noting that His15 is located on motif 10 and factoring in its functional importance to CA-II, data suggests that the potential instability and greater conformational sampling observed in K18E and K18Q could be as a result of interaction loses with His15. The potential instability and greater conformational sampling associated with His15, may however have minimal effect on the catalytic function of K18E and K18Q. Previous enzyme kinetic research performed in 1988 by Eriksson [25] showed that even after removal of the first 23 N-terminal residues, CA-II maintained function and hydrated CO${}_{2}$ at rate constant of 1.5 × 10${}^{5}$ s${}^{-1}$ [25].

Analyzing the contacts of H107Y in Figure 6, results indicate that Tyr107 loses all interactions with Val31. Tyr107 also shows a decrease in interactions with Glu117, and this decline in weighted contacts is greater when the substrates BCT and CO${}_{2}$ are bound to the protein. Active site Zn${}^{2+}$ is coordinated by the direct ligand His119 and the indirect (secondary) ligand Glu117. Residue Glu117 stabilizes the Zn${}^{2+}$ through interactions with the direct ligand His119 [1]. It is observed that Tyr107 reduction in weighted contacts with Glu117 is not as great as that observed with Val31. Due to the importance of Glu117 in Zn${}^{2+}$ affinity, additional interactions could have formed between Tyr107 and Glu117 as a compensatory measure to maintain enzyme structure, function and catalytic efficiency. Potential compensatory mechanisms occurring in H107Y were investigated later in this article using DRN analysis. When CO${}_{2}$ is bound to H107Y, Tyr107 creates new interactions with residue Ala247, as Ala247 is located on motif 3 (Table 2) and formation of new interactions could assist with stability.

**Figure 7:**
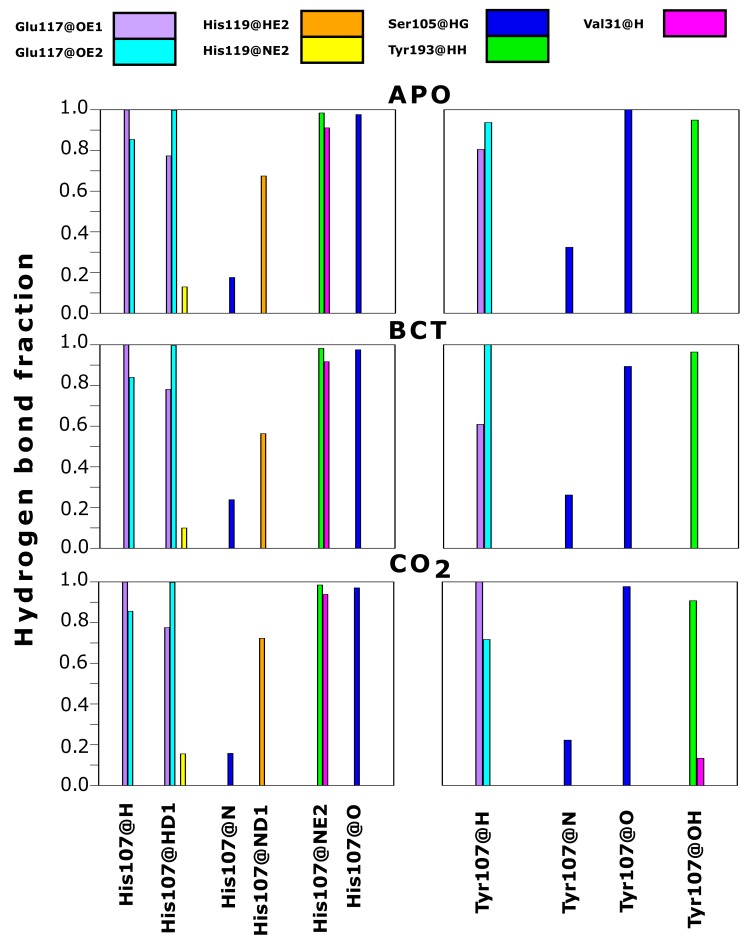
Hydrogen bond fraction between residue 107 and neighboring atoms in the WT and variant proteins. Fraction represents proportion of the total MD simulation frames the hydrogen bond existed between the specific atoms. **Left:** WT. **Right:** H107Y. Suffix **@** refers to residue atoms; **H:** hydrogen; **HD:** delta hydrogen; **HE:** epsilon hydrogen; **HG:** gamma hydrogen; **HH:** eta hydrogen; **N:** nitrogen; **ND:** delta nitrogen; **NE:** epsilon nitrogen; **O:** oxygen; **OE:** epsilon oxygen; **OH:** hydroxyl.

As contact maps do not discriminate between bond/interaction types, hydrogen bond analysis was performed to analyze changes occurring to interactions between residue 107 and neighboring residues. Hydrogen bonds are one of the stronger interactions occurring in proteins, and loss would have significant effects to enzyme stability and structure. Results in Figure 7 compare hydrogen bond fractions (proportion of total MD frames bonds were present) between residue 107 and neighboring atoms in the WT and variant protein. Results show the loss of hydrogen bonds between Tyr107 and the neighboring residues; Val31, Glu117 and His119. One hydrogen bond is lost between Tyr107 and Val31, whereas two hydrogen bonds each are lost between Tyr107 and the residues Glu117 and His119. From data, it is evident that H107Y loses half of the hydrogen bonds present in the WT. Residue 107 in the WT makes 10 hydrogen bonds within neighboring atoms over MD, whereas in the variant, only 5 hydrogen bonds are made with neighboring atoms. Previous studies into H107Y have also proved that at least two hydrogen bonds are lost between Tyr107 and Glu117 [32,73,74,75]. The loss of these hydrogen bonds was identified as being responsible for active site distortion and protein misfolding. In addition, studies in 1991 by Venta et al. [75] demonstrated hydrogen bond loss between Tyr107 and Tyr193 in H107Y. However, within this study, no hydrogen bond loss between these two residues was observed.

The coupling of data in Table 2, Table S2 and Figure 7 allows for the identification of H107Y mechanism of action. Hydrogen bond loss with both Glu117 and His119 could destabilize the active site Zn${}^{2+}$ and possibly increase Zn${}^{2+}$ dissociation from the active site and cause instability. Glu117 has already been shown to have influence on Zn${}^{2+}$ rate of dissociation and enzyme activity [1,26,76].

Analysis of P236H contact maps indicates that there are increases to interactions between His236 and Glu238 in the apo and BCT bound protein; however, P236R shows increases to interactions between Arg236 and Glu238 when substrates BCT and CO${}_{2}$ are bound. Differences to interaction loss between P236H and P236R suggest that although these two variants are located at the same position, the mechanism of action differs. Glu238 is not located in any of the identified CA-II motifs and lies between motif 3 and motif 8. Changes to Glu238 interactions could indirectly affect the motif function.

N252D contact map analysis indicates decreases in weighted interactions between Asp252 and Glu26 for the apo and BCT bound protein. When CO${}_{2}$ is bound, interactions with Glu26 are completely lost. The interaction losses with Glu26 appear to have minimal effect on N252D stability as evidenced by its RMSD distribution.

### 2.8. Dynamic Residue Networks Show Changes in Residue Accessibility and Communication within CA-II

We have identified the direct effects to residue interactions occurring as a result of SNV presence in CA-II and possible consequences to enzyme structure and function. However, results have also suggested potential indirect variant mechanisms of action (i.e., allosteric effects) evidenced by motif analysis (Table 2) and RMSF (Figure 5). To fully understand SNV effects, investigation into indirect and/or compensatory variant mechanisms of action was performed using the average shortest path (*L*) and *betweenness centrality* (*BC*).

#### 2.8.1. Average Shortest Path (*L*)

**Figure 8:**
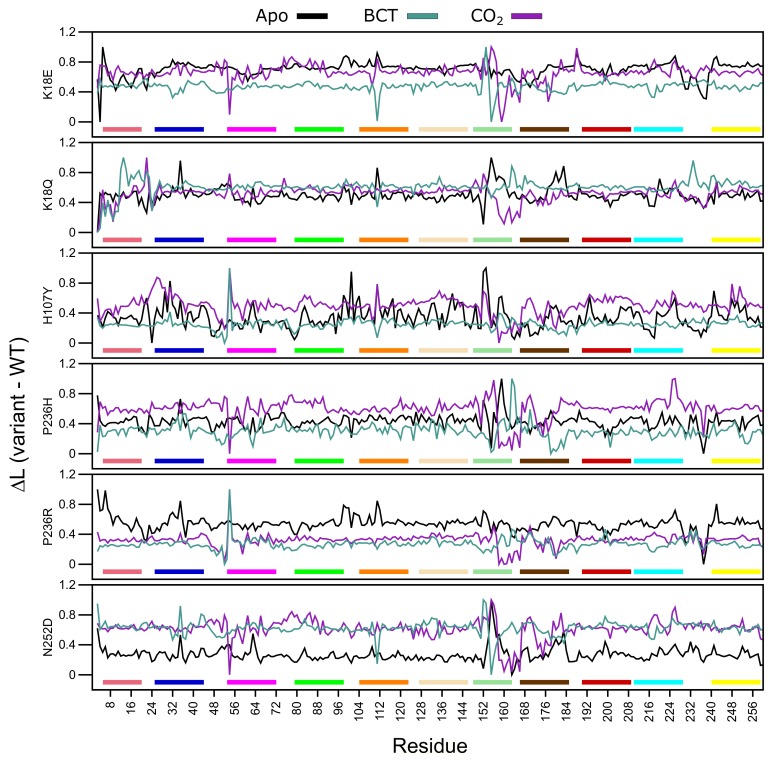
Change in average shortest path *L* between the WT and variant protein (variant minus WT) over the MD simulation. Respective motifs are indicated as bars at the bottom of each plot. Motif colors correspond to those in Figure 2B.

*L* measures the accessibility of a residue within a protein, while average *L* refers to the mean protein residue accessibility across all MD frames. Normalized average Δ*L* (variant minus WT) values were calculated for each WT and variant protein system across all MD frames (Figure 8). A decrease to Δ*L* indicates that variant residues are moving closer to each other with respect to the WT and becoming more accessible, whereas, an increase to Δ*L* indicates a decrease in residue accessibility within the variant in comparison to the WT. In general, Δ*L* calculations identified subtle differences. Table S6 presents residues with an average Δ*L* greater or less than 2 standard deviations from the mean Δ*L* for each protein system indicating significant changes to accessibility.

Overall, in almost all proteins, an increase to residue accessibility was observed for the region of residues ≈150–180 (motif 7 and motif 11), and this trend was also observed within the RMSF results. Previous studies involving *L* and RMSF have suggested that a linear correlation exists between the two metrics [65]. Figure 8 also presents changes to accessibility of Ala54 in all variants. K18Q, H107Y and P236R show decreases to Ala54 accessibility while the other variants do not.

The K18E apo protein shows an increase to residue accessibility in the motif 10 residues His10 and Trp16. With regards to the BCT and CO${}_{2}$ bound K18Q proteins, results show increases to residue accessibility between residues 3 and 11. As motif 10 contains initial aromatic cluster residues (Trp5, Trp7 and Trp16), increases to motif 10 accessibility could explain the greater conformational sampling observed in the RMSD results for K18E apo and BCT and CO${}_{2}$ bound K18Q (Figure 3). The apo protein for K18E, P236H and P236R shows increases to residue accessibility between residues 230 and Glu238. Δ*L* decreases in P236H could be as a result of an increase in contacts between residue 236 and Glu238 observed within the contact maps.

Results in Table S6 indicate that the majority of changes to residue accessibility occur away from residues previously identified as important for CA-II structure and function (Table S1). It should however be noted that although changes to Δ*L* do not occur at these residues, accessibility changes to neighboring residues could still affect important residues as proteins function as a network.

#### 2.8.2. *Betweenness Centrality* (*BC*)

Average *L* allowed for the determination of residues accessibility within the WT and variant proteins. The effect of residue accessibility on protein communication was then measured using *BC*. During MD simulation, protein residues are constantly communicating with one another, and *BC* allows for identification of the most important residues for protein structure and function. Residues with the highest *BC* are most important for protein communication. When SNVs are present, residue communication could change, and analysis of these changes would allow for the identification of compensatory mechanisms employed by variants in an attempt to maintain protein function and stability [41,77].

**Figure 9:**
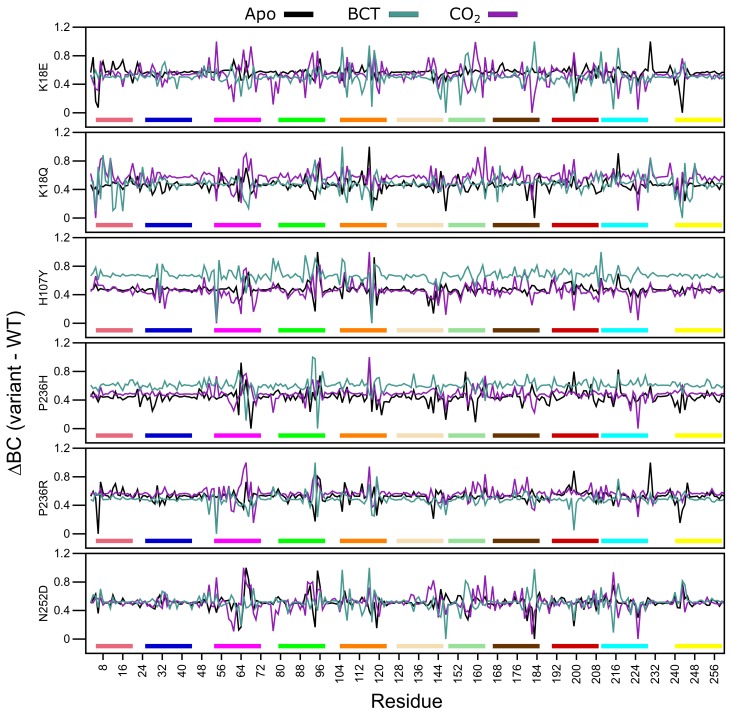
Change in *betweenness centrality* (*BC*) between the WT and variant protein (variant minus WT) over the MD simulation. Respective motifs are indicated as bars at the bottom of each plot. Motif colors correspond to those in Figure 2B.

Average *BC* analysis (data not shown) revealed that Glu117 has the highest BC in all WT and variant proteins. This suggests that Glu117 is the most important residue for communication in CA-II. To observe the *BC* differences between the WT and variant proteins, normalized Δ*BC* (variant minus WT) was calculated. A decrease to Δ*BC* indicates a decrease in residue usage within the variant, whereas an increase to Δ*BC* demonstrates increased residue usage. Figure 9 presents the average normalized Δ*BC* of the WT and variant proteins, and Table S7 shows residues with an average Δ*BC* greater or less than 2 standard deviations from the mean Δ*BC* for each protein system indicating significant changes to residue communication.

K18E apo and K18Q with BCT and CO${}_{2}$ bound demonstrate decreases to Trp5 Δ*BC*. As Trp5 is part of the initial aromatic cluster, the decreased residue usage could explain the greater conformational sampling observed within the RMSD. Analysis of H107Y Δ*BC* results indicates a reduction to the usage of Glu117 for all three protein states. The reduction in usage of Glu117 can be explained by loss of weighted contacts and hydrogen bonds observed. The results further suggest that H107Y could influence Zn${}^{2+}$ dissociation through changes to interactions with Glu117. When CO${}_{2}$ was bound to H107Y, Zn${}^{2+}$ ligand His96 showed an increase in Δ*BC* highlighting a potential compensatory measure to maintain Zn${}^{2+}$ affinity and stability within the active site. More Δ*BC* increases to secondary aromatic cluster residues were observed in the apo and CO${}_{2}$ bound proteins, suggesting potential greater variant effects. In all three H107Y protein systems (apo, BCT and CO${}_{2}$), increases to Δ*BC* of Phe95 were noted. Phe95 is part of motif 4 and is a member of the secondary aromatic cluster. Increases to Phe95 Δ*BC* could be a compensatory measure by H107Y to maintain structure and function.

Within the other variant proteins, the presence of BCT is associated with a reduction in Glu117 usage, highlighting at potential effects on Zn${}^{2+}$ affinity for the active site. In all protein systems Glu117 Δ*BC* decreases are associated with Δ*BC* increases to either of the Zn${}^{2+}$ primary coordinating residues His94, His96 and/or His119 and the Zn${}^{2+}$ secondary ligand Asn243 evidenced in Table S7, highlighting potential compensatory mechanisms in maintaining Zn${}^{2+}$ within the active site. An interesting result is also observed where a decrease in Δ*BC* of one or more Zn${}^{2+}$ coordinating residues is associated with an increase in Δ*BC* of another coordinating residue. For example, Δ*BC* decreases in His94 and His119 in the P236R and N252D apo proteins are associated with Δ*BC* increases to His96. With regards to P236R apo protein the increase to His96 communication could be a result of the decrease in the usage of Asn243. Asn423 is a secondary Zn${}^{2+}$ coordinating ligand that maintains Zn${}^{2+}$ stability through direct interactions with His96 [1].

Δ*BC* results indicate that apart from H107Y, variant effects occur away from the SNV site, and variant presence has allosteric effect on residues important for the structure and function of CA-II as highlighted in Table S7 data. Although SNV effects are more evident at active site residues, some indirect effects are observed at aromatic cluster residues as observed in N252D. This suggests potential implications for precision medicine related studies in the treatment of CA-II deficiencies. Treatment options would have to be targeted to either rescue the primary and secondary aromatic cluster residues or the active site residues.

### 2.9. Variant Presence Shows Remote Effects on Proton Shuttle Residue

As DRN analysis demonstrated evidence that variant effects occur away from the SNV site, the proton shuttle residue His64 was investigated for potential variant effects occurring during protein dynamics that may not have been observed through MD and/or DRN analysis. His64 behavior is also governed by the pKa of surrounding residues [78,79,80] therefore changes to His64 conformations may not be visible using RMSF analysis, and the resulting conformations may not change residue accessibility or usage.

**Figure 10:**
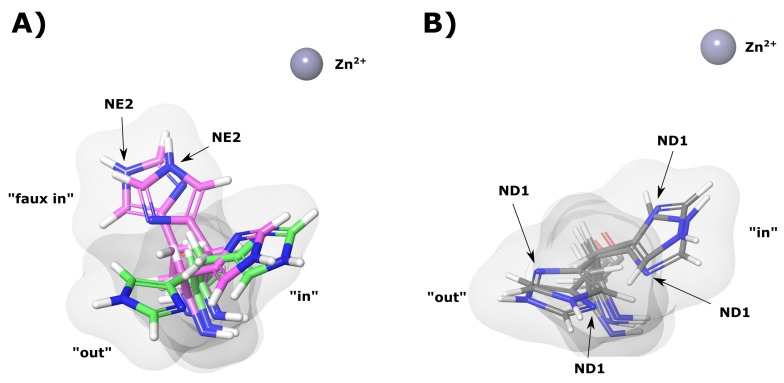
CA-II apo protein His64 “in”, “out” and “faux in” conformations. (**A**) WT and K18E proteins. Green and magenta colors represent WT and K18E, respectively. (**B**) N252D variant. The grey sphere represents the Zn${}^{2+}$, white, blue and red structures represent hydrogen, nitrogen and oxygen atoms, respectively.

During MD simulation His64 was observed to rotate between two main conformations (“in” and “out”). Figure 10A shows an example of these conformations in the WT apo (green) protein. The presence of the two conformations agrees with previous literature findings [16,17,18,19]. Data in Table 3 shows the average distances of the imidazole ring of His64 from the Zn${}^{2+}$ for the “in” and “out” conformations of the majority representative structural clusters. It is evident that the apo proteins are associated with greater His64 conformations.

From the K18E apo results, it is observable that His64 does not occupy an “out” conformation. The variant, however, occupies what we have termed a “faux in” conformation (Figure 10A), which is being observed for the first time in this study, and has not been noted in previously studied literature [1,6,10,16,81]. The “faux in” conformation was observed for a fraction of 0.972 in all MD frames, and interestingly compared to the other protein systems, this conformation brings the imidazole ring closest to the Zn${}^{2+}$. As no “out” conformation for His64 was observed, data suggests that His64 in K18E may not be able to adopt an “out” conformation that could significantly impact proton shuttling as a result of a larger water network being required to shuttle protons out of the active site [82,83,84]. His64 in the “faux in” conformation may also not be able to assist with the stabilization of active site water network to the same extent as the “in” conformation, which could have an effect on reaction rates [83].

K18Q results when BCT is bound (Table 3) show that His64 did not occupy the “out” conformation and remained in the “in” conformation for the duration of the MD simulation. Data in Table 3 also shows an unusual result with regards to P236R apo results. His64 can occupy all three conformations. The “in”, “out” and “faux in” conformations were present for a fraction of 0.049, 0.123 and 0.827 of all MD frames, respectively, suggesting a strong preference for “faux in”. Analysis of the trajectory (results not shown) and the order of emergence of conformations in the MD frames suggests that His64 when in the “faux i” conformation may not be able to rotate directly to the “out” conformation, without transitioning through the “in” conformation initially. With respect to K18E and P236R, the “faux in” conformation was not observed when substrate was bound, suggesting that substrate presence could influence the behavior of His64.

Additional analysis of the “in” and “out” conformations of His64 and other structural clusters also showed another interesting finding in which rotation of the His64 CB-CG (beta carbon atom and gamma carbon atom) bond was observed. An example of this bond rotation is shown in Figure 10A,B evidenced by the NE2 and ND1 atoms of His64 occupying different spatial orientations in K18E and N252D. Rotation of the His64 CB-CG in addition to being observed in the variants was also noted in the WT proteins. This result could suggest potential implications for the mechanism of action of His64 in proton shuttling. Previous research has suggested that either His64 “in” and “out” rotation and/or imidazole tautomerization to be responsible for proton shuttling [16,17,18,19,20]; however, this finding could suggest that in addition to tautomerization of His64 in the “out” conformation to shuttle protons, the His64 CB-CG bond rotation could facilitate imidazole ring rotation to shuttle a proton to and from the active site during catalysis. Additional research is however required to confirm this.

## 3. Materials and Methods

**Figure 11:**
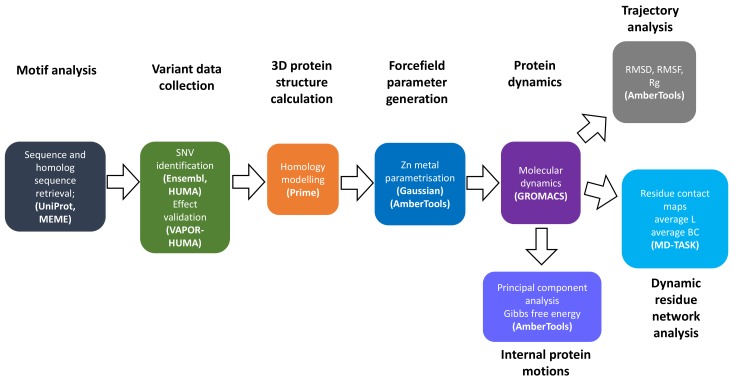
Flow chart of methodology used in analysis of the effects SNVs on CA-II structure and function.

Figure 11 summarizes the overall approach, methods and software used to conduct research in this article.

### 3.1. Data Retrieval

The amino acid sequence of CA-II was retrieved from the Universal Protein Resource (UniProt) [11]. UniProt BLAST was then used to identify other CA sequences within the α-CA family using the CA-II sequence as the query. The BLASTp program was used to search for homologous sequences against the UniProtKB target database using the BLOSUM-62 matrix [85] and the UniProt E threshold search parameter value of 100. From the BLAST results, all human CA proteins CA-I to XIV, including mouse CA-XV, were selected to create the final dataset (Table S2).

### 3.2. Motif Analysis

Discovery and analysis of motifs was performed according to methodology by Ross et al. and Nyamai and Tastan Bishop [56,86] with slight modification using the retrieved sequences as a query. Briefly, motif discovery was performed using online MEME SUITE version 5.05 [55], using the 0-order model of sequences. A minimum and maximum motif width of 5–20 residues was set. A total of 100 motifs for each human α-CA protein were set up for discovery. Discovered MEME motifs were then validated using MAST and E-value for inclusion or exclusion. Only motifs with an E-value less than 0.001 were retained. In addition, motifs with pairwise correlations greater than 0.6 were removed using MAST to create the final dataset. If the pairwise correlation is greater than 0.6, this may cause some *p*-values and E-values to be underestimated and diminishes accuracy [55]. The existence of each final dataset motif was then checked for in each CA sequence, and matplotlib [87] was used to construct a heat map representing motif conversation as the number of sites per total number of protein sequences. Motif logos showing the conservation of each amino acid residue in each motif sequence were also downloaded from the MEME server and visualized using Inkscape [88].

### 3.3. Homology Modeling

#### 3.3.1. Wild-Type

There are 652 entries in the Protein Data Bank (PDB) [89] for the 3D structure of CA-II protein (UniProt accession number: P00918; accessed 20/06/2018); however, the majority of them are missing residue number 126 [90], hindering accurate CA-II SNV modeling, parametrization and MD. Residue 126 exists only in CA-I [91]; therefore, template numbering proceeds from 125 and continues to 127 even though the FASTA sequence matches the ATOM sequence 100%. Some of the structures also lack Zn${}^{2+}$ in the correct coordination geometry, which would have implications on metal site parametrization.

Potential templates from the 652 structures were filtered using AmberTools17 [58] by observing all atoms bonded to the Zn${}^{2+}$ within a radii of 2.5 Å in a tetrahedral geometry. The 2.5 Å is the maximum Zn-ligand bond distance [60,63]. PDBs containing an HHHX (3 Histidines and 1 H_2_O) coordinating formation with Zn${}^{2+}$ were considered, and the best template was selected based on 3D structure resolution and PDB validation. Crystal structure 2VVA with resolution of 1.56 Å, and a sequence similarity of 99% to the UniProt protein (covering residues 3–260) was chosen as the template. 2VVA also contains CO${}_{2}$ co-crystallized to the primary pocket of CA-II. Due to errors encountered when using *pdb4amber* with 2VVA, renumbering was insufficient to correct missing residue 126, and therefore, homology modeling was necessary.

Clustal Ω (Omega) [92,93] was utilized to align the target CA-II sequence (UniProt accession: P00918) and template sequence (2VVA). Schrödinger Prime [52,53] was used to generate five homology models that included the ligands Zn and CO${}_{2}$. Wild-type (WT) model quality was validated using the z-DOPE score and Ramachandran plot. If the z-DOPE score is less than −1.0, homology models are regarded as being like native structures. A Ramachandran plot allows for the identification of energetically favorable amino acid backbone conformations by comparison of φ (phi) and ψ (psi) torsion angles.

#### 3.3.2. Variants

SNV analysis was carried out according to proposed protocol by Brown and Tastan Bishop, 2017 [94]. Briefly, to the downloaded SNVs from HUMA [44] and Ensembl [43], datasets were filtered to include only CA-II nsSNVs. Datasets were further filtered to include only SNVs validated within dbSNP [95]. SNVs were then cross referenced with the Clinvar [96] and OMIM [97] databases to isolate pathogenic variants. VAPOR in HUMA [44] was used to predict the expected effects of these SNVs on CA-II structure and function.

Homology modeling was performed to introduce the identified nsSNVs into the CA-II structure, with unique structures generated for each nsSNV. The CA-II amino acid sequence was first modified to contain the required SNVs. Homology modeling was then performed according to the WT methodology. The WT protein model was used as the template, and five models including all HETATMs (hetero-atoms) were generated per variant protein. The z-DOPE score and Ramachandran plots were used to validate the quality of the models.

WT and variant apo protein models were generated by removing CO${}_{2}$ from the modeled proteins.

#### 3.3.3. Bicarbonate Bound Structure

Besides the WT and variant apo and CO${}_{2}$ bound protein models, bicarbonate (BCT) bound WT and variant complexes were also calculated using the crystal structure (PDB ID: 2VVB). In this case, the apo WT and variant protein models were superimposed with 2VVB using PyMOL [98]. The coordinates of BCT were then added to the apo proteins to generate the final BCT containing structures. Superposition to incorporate BCT into the protein structure was preferred as opposed to modeling. The superposition allows for the maintenance of one reference structure (CO${}_{2}$ bound model) for apo, CO${}_{2}$ and BCT bound structures comparison. Modeling could have introduced some structural changes to the proteins and inhibited direct comparison of all three protein models for WT and variant proteins.

In total, 21 models (7 apo, 7 with CO${}_{2}$ and 7 proteins with BCT) were calculated and taken into the further calculations as explained below.

### 3.4. Zn${}^{2+}$ Parametrization

Due to the presence of Zn${}^{2+}$ in the CA-II active site and its importance in protein function, AmberTools17 was used to perform metal site parametrization for the 21 WT and variant CA-II protein models, utilizing the bonded model approach and the MCPB [62]. Zn${}^{2+}$ parametrization was necessary to develop atom forcefields for interactions and to ensure the metal remained within the active site during MD simulations.

CA-II protein structures where protonated using the H++ server [99] at pH 7.0 [100,101], with an internal and external dielectric of 10 and 80, respectively, and a system salinity of 0.15 M. AMBER topology and coordinate files (*top* and *crd*) were then downloaded from the server, and *ambpdb* used to generate a protonated PDB file. To the generated PDB file, protonation states of all structures were then validated by ensuring that the Zn${}^{2+}$ coordinating ligands; His94, His96 and His119 were in the correct protonation states (HID, HID, and HIE, respectively).

MCPB was then used to generate Gaussian 09 input files, prior to QM calculations. QM calculations were performed according to methodology by Li and Merz 2017 [62] using the B3LYP/6-31G basis set in Gaussian 09 [59], using 192 CPU cores at the Center for High Performance Computing (CHPC) cluster, Cape Town South Africa. To the Gaussian 09 output files, MCPB was then used to calculate bond lengths, angles and dihedrals between Zn${}^{2+}$ and coordinating atoms, to derive the final Zn${}^{2+}$ forcefield parameters.

### 3.5. Molecular Dynamics

For MD simulations, CA-II protein structures were protonated by the Schrödinger Maestro [102] Protein Preparation Wizard in conjunction with PROPKA [103] at pH 7.0 [100,101]. The previously generated forcefield parameters were used in conjunction with Leap modeling [104] to generate AMBER topologies, utilizing the AMBER ff14SB forcefield [105] and a cubic box of cut-off distance 10 Å (distance between protein molecule and box). To the box, water molecules adhering to the TIP3P water model were added as solvent, and a concentration of 0.15 M NaCl was used to neutralize the system. Generated AMBER topology files were then converted to GROMACS [106] topology using ACPYPE [107] to generate *gro* and *top* files, prior to energy minimization. Generated topology files were then manually inspected to ensure accurate neutralization by comparing the total protein charge (*qtot*) to the quantity of counter-ion added. AMBER to GROMACS topology conversion, using ACPYPE, maintains all previously set Leap parameters, such as cubic box cut-off distance, across the different programs, and generated topologies can be directly minimized.

MD simulations were conducted for all 21 protein structures using GROMACS 2018.2 [106]. Energy minimization was setup for 50,000 steps using the steepest descent algorithm and terminated when a maximum force (F${}_{max}$) of no greater than 1000 kj mol${}^{-1}$ nm${}^{-1}$ was attained, and the system converged. Temperature and pressure equilibration (*NVT* and *NPT* ensemble, respectively) were performed after energy minimization, using the modified Brenson thermostat and the Particle Mesh Ewald (PME) coulomb type for long-range electrostatics. All bonds were constrained under the LINCS algorithm. The *NVT* ensemble was performed for 100 ps at 300 K, followed by the NPT ensemble, until the system stabilized at a pressure of 1 bar. MD simulations were performed at the CHPC cluster, on one Nvidia Tesla v100 GPU in conjunction with 10 CPU cores over a period of 200 ns, with a time integration step of 2 fs. Coordinates were written to file every 10 ps.

### 3.6. Molecular Dynamics Trajectory Analysis

To ensure efficient and accurate protein analysis, the trajectories resulting from the MD simulations were stripped of all periodic boundary conditions and centered within the simulation box using *cpptraj* [108]. From the corrected trajectory, new PDB files for the WT and protein variants were also generated for MD analysis. The Visual Molecular Dynamics program (VMD) [109] was used to visualize the trajectory and to evaluate whether the system adhered to the parameters set and whether the Zn${}^{2+}$ remained in place.

The AmberTools17 package *cpptraj* was used to calculate RMSD, RMSF and the Rg of the protein α-carbons.

### 3.7. Statistical Analysis

Statistical analysis of the RMSD and the Rg data was performed using the z-test for parametric data and the Mann–Whitney U test (MWU) for non-parametric data. All statistical analysis and data generation was performed using the RStudio v1.1.456 [110] integrated development environment, in combination with R v3.5.1 [111].

### 3.8. Proton Shuttle Analysis

To observe changes to proton shuttle residue behavior during MD simulation, structural clustering based on His64 conformations was performed for all protein systems using the hierarchical agglomerative (bottom up) algorithm and the average-linkage method [112,113,114], in conjunction with *cpptraj* and the *cluster* command. Cluster command was set to perform clustering every four frames to generate a total of four protein clusters to account for His64 “in” and “out” conformations, flips in the imidazole ring and other potential orientations.

Conformational clusters were generated using the following three criteria: angle between His64 CB, CG atoms and the Zn${}^{2+}$; dihedral angles between His64 N, CA, CB and CG atoms (chi1) and CA, CB, CG and ND1 (chi2); and distance between His64 ND1 atom and the Zn${}^{2+}$. Protein structures representative of each conformational cluster were also generated.

### 3.9. Dynamic Residue Network Analysis

DRN analysis for each protein was done using MD-TASK [42]. Residue interaction was predicted through the evaluation of pairwise distances between all Cβ (Cα for glycine) atoms, across all complexes present in each frame during the MD trajectory. Within the DRN, each protein residue is a node within the network [42].

#### 3.9.1. Weighted Contact Map Analysis

Contact maps show the frequency of interaction between two residues over an MD trajectory. Residue–residue interactions in the WT and protein variants over the 200 ns simulations were calculated using the *contact_map.py* script in MD-TASK, using a cut-off distance of 6.7 Å previously reported for Cα-Cα node interaction [115]. The weighted contacts of the WT and SNV residues were compared to observe the effect of SNVs on short-range residue–residue interaction.

#### 3.9.2. Average Shortest Path (L)

*L* measures the accessibility of a protein node (specific residue) by computing the total number of shortest paths to that node and dividing by the total number of nodes minus one [42]. Equation (2) is used to calculate *L*.

(2) $$\alpha =\sum_{s,t\in V}\frac{d(s,t)}{n(n-1)}$$

From Equation (2), *V* is the set of nodes in the network. d(s,t) is the shortest path from *s* to *t*, while n represents the total number of nodes within the network [116]. An increase in *L* shows a decrease in residue accessibility, while decreases in *L* signify an increase in residue accessibility. *L* was calculated across all MD frames for a threshold of 6.7 Å using the *calc_network.py* script in MD-TASK. Calculated *L* was then normalized on a scale of 0 to 1 using unity-based normalization to generate normalized *L*. The average normalized *L* was determined by averaging normalized residue *L* across all frames of trajectory for each residue in each protein. The Δ*L* for each residue in the DRN was then calculated by subtracting the average normalized *L* of the WT and variant proteins (variant minus WT).

#### 3.9.3. Average *Betweenness Centrality (BC)*

*BC* governs the importance of a residue for protein communication. The *BC* of a node equates to the number of shortest paths passing through that node, interlinking one specific node to numerous others. *BC* is determined according to Equation (3).

(3) $$C_{B}\left(v\right)=\sum_{s,t\in V}\frac{\sigma (s,t|v)}{\sigma (s,t))}$$

Within Equation (3), *V* represents network nodes, whereas σ(s,t) is the number of shortest (s,t) paths. σ(s,t|v) is the number of those paths passing through node v other than s,t [116]. The more frequent the communication of a residue within a protein, the higher the *BC*, indicating the importance of that residue within the protein. *BC* was determined following methodology to that of *L* (Section 3.9.2). Briefly, *BC* for each protein residue was calculated using the *calc_network.py* script for a threshold of 6.7 Å. Unity based normalization was applied to generate normalized *BC*. The average normalized *BC* for each protein residue was then determined by averaging the normalized *BC*. WT and variant average normalized *BC* were then subtracted (variant minus WT) to calculate Δ*BC*.

### 3.10. Principal Component Analysis (PCA)

PCA was performed using *cpptraj* according to methodology in 2014 by Roe et al. [117]. Global rotational/translational motion was removed from the trajectories by applying an RMS best-fit to an average structure. The coordinate covariance matrix was then calculated for all heavy atoms (excluding hydrogen) for the WT protein and variants, followed by diagonalizing to obtain the eigenvectors and eigenvalues. Variant coordinates were then projected along each eigenvector to obtain separate projections for each trajectory set. The first and second projections where then normalized and plotted against each other to obtain a graph of PC1 against PC2 using the *hist* analysis command of *cpptraj*. The free energy associated with PC1 and PC2 at 300 K was also calculated using the *hist* command.

## 4. Conclusions

In conclusion, motif analysis, MD, PCA and DRN techniques were used to investigate the potential effects of the six validated SNVs (K18E, K18Q, H107Y, P236H, P236R and N252D) on the structure, stability and function of human α-CA-II in both the presence and absence of substrates BCT and CO${}_{2}$. The study was divided into two parts comprising global and local level protein analysis. Motif studies identified 11 motifs potentially important to CA-II structure, function and stability. Only the variants K18E, K18Q, H107Y and N252D are, however, located on these motifs. Global level analysis indicated subtle effects of variant presence on CA-II structure. The presence of substrates BCT and CO${}_{2}$ is associated with greater SNV effects, and in general, when substrate BCT is bound, PCA analysis showed an increase to conformational sampling and free energy in all protein structures. Local analysis using DRN demonstrated that the effects of the SNVs on CA-II structure and function occur away from the SNV location, indicating an allosteric/indirect SNV effect. In all cases, Glu117 was identified as the most important residue for communication within CA-II. Variant H107Y results showed a reduction to interactions between Tyr107 and Glu117. A loss of at least two hydrogen bonds was also noted for these two residues. Average *BC* analyses indicated a reduction to the usage of residue Glu117 in H107Y for all three protein states as a result of the interaction loss, while the other variants showed a reduction to Glu117 usage mainly when BCT was bound. The reduction to Glu117 usage suggests potential implications for metal ion affinity for the CA-II active site and was associated with increases in the usage of the Zn${}^{2+}$ coordinating residues His94, His96 and His119, indicating compensatory mechanisms to maintain Zn${}^{2+}$ within the active site. Proton shuttle analysis also highlighted the formation of a novel His64 conformation “faux in” in K18E and P236R apo proteins. The imidazole ring of the "faux in" conformation is located closer to the Zn${}^{2+}$ compared to the “in” and “out” conformations.

In addition to the findings summarized above, we also emphasize that MD simulations coupled with DRN analysis [41,42] would provide detailed insights into the understanding of SNV mechanism of action at the molecular level and would help with precision medicine related studies. In light of our findings, this study proposes taking steps towards the treatment of CA-II deficiencies, which would either be by rescuing the primary and secondary aromatic cluster residues or the active site residues. Possible future work could include quantum mechanical calculations as to the effect of SNVs on the proton transfer pathways in CA-II.

## Figures and Tables

**Table 1:** α-CA-II single SNVs rs IDs, associated residue-variant substitutions, model z-DOPE score, MAF and predicted variant consequences. Global MAF obtained from gnomAD.

rs ID	Variation	z-DOPE Score	MAF	VAPOR Analysis			
				I-Mutant		MUpro	
				ΔΔG	Stability	ΔΔG	Stability
**rs118203931**	K18E	−2.204	<0.01	−1.30	Decrease	−0.499	Decrease
	K18Q	−2.203	<0.01	−1.24	Decrease	−0.655	Decrease
**rs118203933**	H107Y	−2.204	<0.01	0.26	Increase	−0.867	Decrease
**rs118203932**	P236H	−2.159	<0.01	−1.46	Decrease	−0.586	Decrease
	P236R	−2.186	<0.01	−0.60	Decrease	−0.311	Decrease
**rs2228063**	N252D	−2.197	0.007	0.06	Increase	−0.474	Decrease

**Table 2:** Starting and ending positions of conserved motifs in CA-II, associated E-values and motif contribution to function. Residues from Table S1 are underlined and highlighted in bold. SNV positions (K18E, K18Q, H107Y and N252D) are underlined, italicized and presented in bold red.

Motif	Residue Range	Residues	E-Value	Residue Count	Contribution to Functions
**1**	190–209	YWTYPGS**LTTP**PLLECVT**W**I	2.2 × 10${}^{-164}$	20	Catalytic mechanism and Primary CO${}_{2}$ binding pocket formation
**2**	104–123	GS**E**H****TVDKKKYAA**E**L**H**L**V**HW	4.4 × 10${}^{-140}$	20	Active site and/or Zn${}^{2+}$ stability
**3**	240–259	MVD**N**W**R**PAQPLK****N****RQIKASF	1.2 × 10${}^{-108}$	20	Tertiary CO${}_{2}$ binding pocket formation and enzyme stability
**4**	79–98	LKGGPLDGTYRLIQ**FHFHW**G	3.0 × 10${}^{-89}$	20	Enzyme stability and/or Zn${}^{2+}$ coordination
**5**	25–44	GERQ**S**PVDIDTHTAKYDPSL	6.5 × 10${}^{-86}$	20	Enzyme stability
**6**	127–146	YGDFGKAVQQPDGLA**V**LGIF	3.8 × 10${}^{-73}$	20	Primary CO${}_{2}$ binding pocket formation
**7**	166–185	IKTKGKSAD**F**TN**F**DPRGLLP	6.0 × 10${}^{-54}$	20	Participated in secondary aromatic cluster
**8**	210–229	VLKEPISVSSEQVLK**F**RKLN	6.4 × 10${}^{-40}$	20	Enzyme stability and secondary CO${}_{2}$ binding pocket formation
**9**	53–72	QATSLRILN**N**G**H**A**FN**VE**F**DD	1.9 × 10${}^{-41}$	20	Enzyme stability and/or catalytic mechanism
**10**	5–20	**W**G**Y**GKHNGPEH**W**H****K****D**F**	7.7 × 10${}^{-25}$	16	Enzyme stability
**11**	148–163	KVGSAKPGLQKVVDVL	2.3 × 10${}^{-6}$	16	Enzyme stability

**Table 3:** Distance of His64 imidazole group from Zn${}^{2+}$ for the “in” and “out” conformations. All distances are measured from the His64 imidazole ring centroid to the Zn${}^{2+}$. Faux refers to other conformations observed excluding traditional “in” and “out” occupied by His64.

Variant	Imidazole-Zn${}^{2+}$ Distance (Å)								
	apo			BCT			CO${}_{2}$		
	In	Out	Faux in	In	Out	Faux in	In	Out	Faux in
**K18E**	8.65	*	7.30	8.08	11.09	*	6.96	11.20	*
**K18Q**	8.11	11.01	*	8.22	*	*	8.96	10.42	*
**H107Y**	8.50	10.63	*	8.24	10.86	*	7.98	10.67	*
**P236H**	8.57	11.26	*	8.70	12.02	*	7.12	11.71	*
**P236R**	8.26	11.02	7.36	7.99	10.84	*	7.50	10.43	*
**N252D**	8.11	11.33	*	8.65	10.93	*	8.63	11.20	*
**WT**	8.24	11.21	*	8.57	11.07	*	8.45	11.31	*

* Conformation not observed.

## Supplementary Materials

The following are available online, Table S1: Identified CA-II residues important for structure, function and stability. Table S2: Table of UniProt CA accession numbers for sequences used in motif discovery. Figure S1: Heat map of all conserved motifs within human α-CA family and associated UniProt accession Motif conservation is represented as number of motif sites per total protein sequences. A value of zero shows that motifs are not conserved in any sequence, whereas a value of 1 shows that motif is conserved in all sequences. The prefix ’M’ represents the word motif. Table S3: Zn${}^{2+}$ non-bonded, bonds, angles and dihedral parameters derived within this study **K${}_{b}$:** bond force constant; **K${}_{\theta }$:** angle force constant; **R${}_{min}$:** vdW radius; **ϵ:** LJ potential well energy. Figure S2: RMSD comparison between the WT and variant proteins during MD. Table S4: Associated *p*-values for the RMSD and Rg distribution data. Figure S4: 3-dimensional (3D) plot of PC1 vs PC2 of the WT and variants proteins as a function of free energy. Free energy is represented in kcal/mol. Table S5: Eigenvalue fraction of each principal component for the active site residues within the wild-type and variant proteins in the absence (apo) and presence (non apo) of CO${}_{2}$. Figure S4: Rg comparison between the WT and variant proteins during MD. Figure S5: Residue RMSF comparison between the WT and variant proteins. Table S6: Variant residues showing decreases and increases to Δ*L* during MD simulation. Residues from Table S1 are underlined and highlighted in bold. SNV positions are underlined, italicized and highlighted in bold red. Table S7: Variant residues showing decreases and increases to Δ*BC* during MD simulation. Residues from Table S1 are underlined and highlighted in bold. SNV positions are underlined, italicized and highlighted in bold red.

## Abbreviations

The following abbreviations are used in this manuscript:

BC	*Betweenness centrality*
BCT	Bicarbonate
BLAST	Basic local alignment search tool
CA	Carbonic anhydrase
DRN	Dynamic residue network
L	Average shortest path
LJ	Lennard–Jones
MCPB	Metal Centre Parameter Builder
MD	Molecular dynamics
PCA	Principal component analysis
QM	Quantum mechanical
RMSD	Root mean square deviation
RMSF	Root mean square fluctuation
Rg	Radius of gyration
SNV	Single nucleotide variation
WT	Wild type
