# Supplementary material for: Mechanism of Action of Non-Synonymous Single Nucleotide Variations Associated with α-Carbonic Anhydrase II Deficiency
Source: Molecules. 2019 Nov 4;24(21):3987. doi: 10.3390/molecules24213987 (PMC6864701; doi:10.3390/molecules24213987)
Supplement: Supplementary file 1 [file molecules-24-03987-s001.pdf]

Supplementary Materials: Mechanism of action of non-synonymous single nucleotide variations associated with α-carbonic anhydrase II deficiency

Taremekedzwa Allan Sanyanga [1], Bilal Nizami [1,2] and Özlem Tastan Bishop [1*]

Table S1. Identified CA-II residues important for structure, function and stability.

Residue	Functional role	Reference[a]
**Trp5**	Primary aromatic cluster residue, tertiary CO_2 binding pocket formation, His64 stabilisation when in the "out" conformation	[1–3]
**Tyr7**	Primary aromatic cluster residue, active site water network coordination	[1–9]
**Trp16**	Primary aromatic cluster residue	[1–3]
**Phe20**	Primary aromatic cluster residue	[1–3]
**Ser29**	Enzymatic stability	[10,11]
**Asn62**	Active site water network coordination	[4]
**His64**	Proton shuttling residue, tertiary CO_2 binding pocket formation	[1,4–8]
**Phe66**	Secondary aromatic cluster residue, secondary CO_2 binding pocket formation	[1,5–8]
**Asn67**	Active site water network coordination	[12]
**Phe70**	Secondary aromatic cluster residue	[1–3]
**Phe93**	Secondary aromatic cluster residue	[1–3]
**Gln92**	Secondary Zn^{2+} ligand	[1,4]
**His94**	Zn^{2+} coordination	[1,4]
**Phe95**	Secondary aromatic cluster residue, secondary CO_2 binding pocket formation	[1,5–8]
**His96**	Zn^{2+} coordination	[1,4]
**Trp97**	Secondary aromatic cluster residue, secondary CO_2 binding pocket formation	[1,5–8]
**Glu106**	Orientation of Zn^{2+} water ligand molecule for catalysis	[1]
**Glu117**	Zn^{2+} affinity and catalytic efficiency, secondary Zn^{2+} ligand	[13]
**His119**	Zn^{2+} coordination	[1,4]
**Val121**	Primary CO_2 binding pocket formation	[1,5–8]
**Val142**	Primary CO_2 binding pocket formation	[1,5–8]
**Phe175**	Secondary aromatic cluster residue	[1–3]
**Phe178**	Secondary aromatic cluster residue	[1–3]
**Leu197**	Primary CO_2 binding pocket formation	[1,5–8]
**Thr198**	Deep water molecule stabilisation, catalytic orientation of Zn^{2+} water ligand molecule	[4]
**Thr199**	Active site water coordination, tertiary CO_2 binding pocket formation	[4]
**Pro200**	Tertiary CO_2 binding pocket formation	[1,5–8]
**Trp208**	Primary CO_2 binding pocket formation	[1,5–8]
**Phe225**	Secondary aromatic cluster residue, secondary CO_2 binding pocket formation	[1,5–8]
**Asn243**	Tertiary CO_2 binding pocket formation, secondary Zn^{2+} ligand	[1,5–8]
**Arg245***	Enzyme stability	[14]

* associated with stability reduction in CA-I

[a] references at end of supplementary data

Table S2. Table of UniProt CA accession numbers for sequences used in motif discovery.

Sequence	UniProt accession
**CA-I**	P00915
**CA-II**	P00918
**CA-III**	P07451
**CA-IV**	P22748
**CA-VA**	P35218
**CA-VB**	Q9Y2D0
**CA-VI**	P23280
**CA-VII**	P43166
**CA-VIII**	P35219
**CA-IX**	Q16790
**CA-X**	Q9NS85
**CA-XI**	O75493
**CA-XII**	043570
**CA-XIII**	Q8N1Q1
**CA-XIV**	Q9ULX7
**CA-XV**	Q99N23

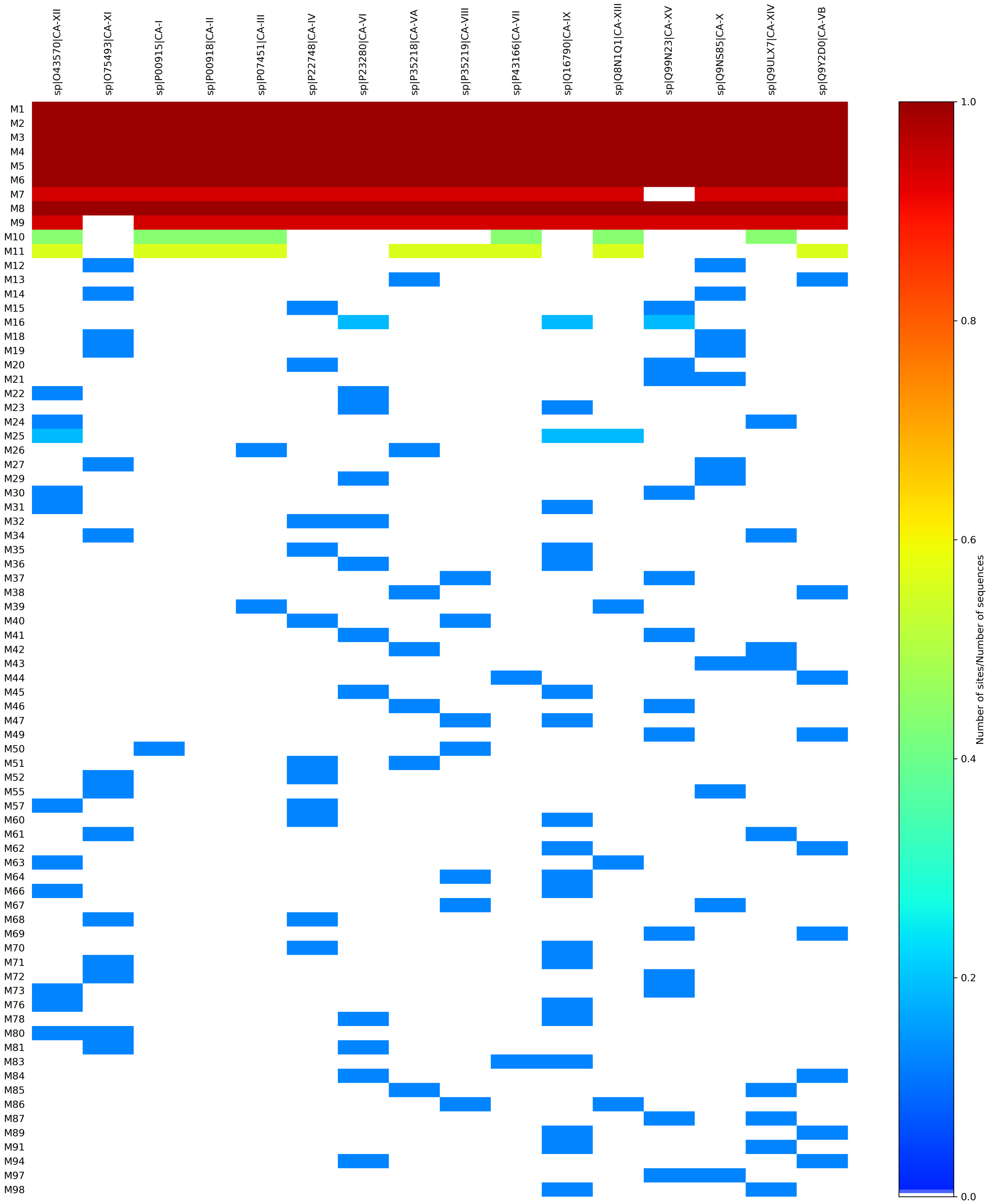

Figure S1. Heat map of all conserved motifs within human α-CA family and associated UniProt accession. Motif conservation is represented as number of motif sites per total protein sequences. A value of zero shows that motifs are not conserved in any sequence, whereas a value of 1 shows that motif is conserved in all sequences. The prefix 'M' represents the word motif.

Table S3. Zn^{2+} non-bonded, bonds, angles and dihedral parameters derived within this study. $\mathbf{K}_b$: bond force constant; $\mathbf{K}_\theta$: angle force constant; $\mathbf{R}_{min}$: vdW radius; ϵ: LJ potential well energy.

Non-bonded:

Atom	R_{min} (Å)	ϵ (kcal/mol)
**M1**	1.40	0.02
**Y1**	1.82	0.17
**Y2**	1.82	0.17
**Y3**	1.82	0.17
**Y4**	1.77	0.15

Bonds:

Bond type	K_b (kcal/mol/Å^2)	Bond length (Å)
**M1-Y4**	41.20	2.12
**Y1-M1**	91.70	1.98
**Y2-M1**	94.30	1.98
**Y3-M1**	93.00	1.98

Angles:

Angle type	K_θ (kcal/mol/radian^2)	Equilibrium angle degrees (θ)
**CC-Y3-M1**	56.44	127.30
**CR-Y1-M1**	38.97	125.70
**CR-Y2-M1**	53.67	127.46
**M1-Y1-CV**	39.62	128.05
**M1-Y2-CV**	54.89	126.22
**M1-Y3-CR**	54.18	125.48
**M1-Y4-HW**	44.55	122.59
**Y1-M1-Y2**	39.89	116.07
**Y1-M1-Y3**	37.44	115.90
**Y1-M1-Y4**	31.02	101.10
**Y2-M1-Y3**	36.29	114.63
**Y2-M1-Y4**	36.83	105.42
**Y3-M1-Y4**	30.04	100.62

Dihedral:

Definition	Divider	Barrier (kcal/mol)	Phase degrees (θ)	Periodicity
**X -CC-Y3-X**	2	4.80	180.0	2.0
**X -CR-Y1-X**	2	10.00	180.0	2.0
**X -CR-Y2-X**	2	10.00	180.0	2.0
**X -CV-Y1-X**	2	4.80	180.0	2.0
**X -CV-Y2-X**	2	4.80	180.0	2.0
**X -Y3-CR-X**	2	10.00	180.0	2.0
**CX-CT-CC-Y3**	1	0.05	180.0	-4.0
**CX-CT-CC-Y3**	1	0.74	0.0	-3.0
**CX-CT-CC-Y3**	1	0.20	0.0	-2.0
**CX-CT-CC-Y3**	1	0.69	0.0	1.0

M1: Zn; **Y1:** His94 NE2 (epsilon nitrogen); **Y2:** His96 NE2 ((epsilon nitrogen)) **Y3:** His199 ND1 (delta hydrogen); **Y4:** O (H_2O); **CC:** CG (gamma carbon); **CR:** CE1 (epislon carbon); **CV:** CD2 (delta carbon)

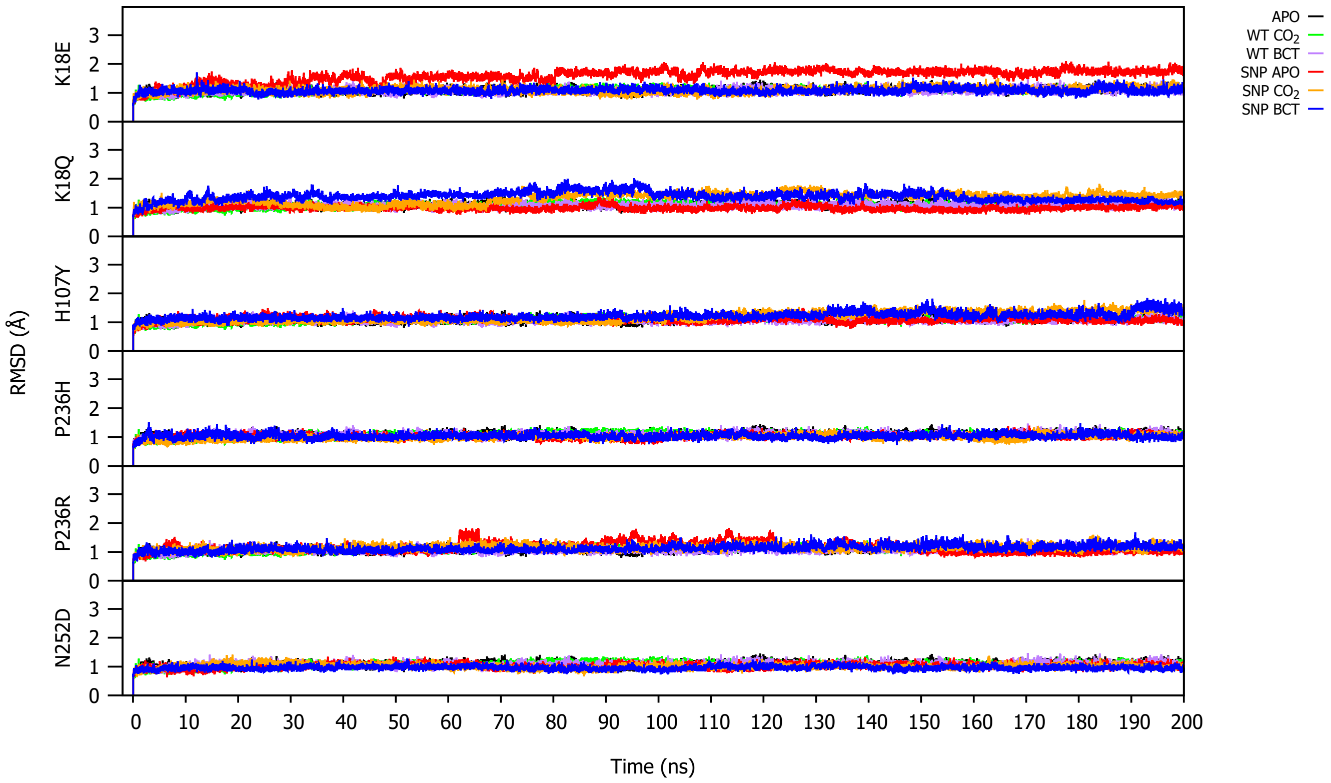

Figure S2. RMSD comparison between the WT and variant proteins during MD.

Table S4. Associated *p*-values for the RMSD and Rg distribution data.

Variant		H107Y			K18E			K18Q			N252D			P236H			P236R			WT		
**Root mean square deviation (RMSD)**																						
		Apo	BCT	CO2	Apo	BCT	CO2	Apo	BCT	CO2	Apo	BCT	CO2	Apo	BCT	CO2	Apo	BCT	CO2	Apo	BCT	CO2
**K18E**	Apo	$< 2.2 \times 10^{-16}$	$< 2.2 \times 10^{-16}$	$< 2.2 \times 10^{-16}$	1	$< 2.2 \times 10^{-16}$	$< 2.2 \times 10^{-16}$	$< 2.2 \times 10^{-16}$	$< 2.2 \times 10^{-16}$	$< 2.2 \times 10^{-16}$	$< 2.2 \times 10^{-16}$	$< 2.2 \times 10^{-16}$	$< 2.2 \times 10^{-16}$	$< 2.2 \times 10^{-16}$	$< 2.2 \times 10^{-16}$	$< 2.2 \times 10^{-16}$	$< 2.2 \times 10^{-16}$	$< 2.2 \times 10^{-16}$	$< 2.2 \times 10^{-16}$	$< 2.2 \times 10^{-16}$	$< 2.2 \times 10^{-16}$	$< 2.2 \times 10^{-16}$
	BCT	$< 2.2 \times 10^{-16}$	$< 2.2 \times 10^{-16}$	$< 2.2 \times 10^{-16}$	$< 2.2 \times 10^{-16}$	1	$< 2.2 \times 10^{-16}$	$< 2.2 \times 10^{-16}$	$< 2.2 \times 10^{-16}$	$< 2.2 \times 10^{-16}$	$< 2.2 \times 10^{-16}$	$< 2.2 \times 10^{-16}$	$< 2.2 \times 10^{-16}$	$< 2.2 \times 10^{-16}$	$< 2.2 \times 10^{-16}$	$< 2.2 \times 10^{-16}$	$< 2.2 \times 10^{-16}$	$< 2.2 \times 10^{-16}$	$< 2.2 \times 10^{-16}$	1.731×10^{-15}	$< 2.2 \times 10^{-16}$	$< 2.2 \times 10^{-16}$
	CO2	0.005176	$< 2.2 \times 10^{-16}$	$< 2.2 \times 10^{-16}$	$< 2.2 \times 10^{-16}$	$< 2.2 \times 10^{-16}$	1	$< 2.2 \times 10^{-16}$	$< 2.2 \times 10^{-16}$	$< 2.2 \times 10^{-16}$	$< 2.2 \times 10^{-16}$	$< 2.2 \times 10^{-16}$	$< 2.2 \times 10^{-16}$	$< 2.2 \times 10^{-16}$	$< 2.2 \times 10^{-16}$	$< 2.2 \times 10^{-16}$	$< 2.2 \times 10^{-16}$	$< 2.2 \times 10^{-16}$	$< 2.2 \times 10^{-16}$	$< 2.2 \times 10^{-16}$	$< 2.2 \times 10^{-16}$	5.517×10^{-07}
**K18Q**	Apo	$< 2.2 \times 10^{-16}$	$< 2.2 \times 10^{-16}$	$< 2.2 \times 10^{-16}$	$< 2.2 \times 10^{-16}$	$< 2.2 \times 10^{-16}$	$< 2.2 \times 10^{-16}$	1	$< 2.2 \times 10^{-16}$	$< 2.2 \times 10^{-16}$	$< 2.2 \times 10^{-16}$	$< 2.2 \times 10^{-16}$	$< 2.2 \times 10^{-16}$	$< 2.2 \times 10^{-16}$	$< 2.2 \times 10^{-16}$	$< 2.2 \times 10^{-16}$	$< 2.2 \times 10^{-16}$	$< 2.2 \times 10^{-16}$	$< 2.2 \times 10^{-16}$	$< 2.2 \times 10^{-16}$	$< 2.2 \times 10^{-16}$	$< 2.2 \times 10^{-16}$
	BCT	$< 2.2 \times 10^{-16}$	$< 2.2 \times 10^{-16}$	$< 2.2 \times 10^{-16}$	$< 2.2 \times 10^{-16}$	$< 2.2 \times 10^{-16}$	$< 2.2 \times 10^{-16}$	$< 2.2 \times 10^{-16}$	1	$< 2.2 \times 10^{-16}$	$< 2.2 \times 10^{-16}$	$< 2.2 \times 10^{-16}$	$< 2.2 \times 10^{-16}$	$< 2.2 \times 10^{-16}$	$< 2.2 \times 10^{-16}$	$< 2.2 \times 10^{-16}$	$< 2.2 \times 10^{-16}$	$< 2.2 \times 10^{-16}$	$< 2.2 \times 10^{-16}$	$< 2.2 \times 10^{-16}$	$< 2.2 \times 10^{-16}$	$< 2.2 \times 10^{-16}$
	CO2	$< 2.2 \times 10^{-16}$	$< 2.2 \times 10^{-16}$	$< 2.2 \times 10^{-16}$	$< 2.2 \times 10^{-16}$	$< 2.2 \times 10^{-16}$	$< 2.2 \times 10^{-16}$	$< 2.2 \times 10^{-16}$	$< 2.2 \times 10^{-16}$	1	$< 2.2 \times 10^{-16}$	$< 2.2 \times 10^{-16}$	$< 2.2 \times 10^{-16}$	$< 2.2 \times 10^{-16}$	$< 2.2 \times 10^{-16}$	$< 2.2 \times 10^{-16}$	$< 2.2 \times 10^{-16}$	$< 2.2 \times 10^{-16}$	$< 2.2 \times 10^{-16}$	$< 2.2 \times 10^{-16}$	$< 2.2 \times 10^{-16}$	$< 2.2 \times 10^{-16}$
**H107Y**	Apo	1	$< 2.2 \times 10^{-16}$	$< 2.2 \times 10^{-16}$	$< 2.2 \times 10^{-16}$	$< 2.2 \times 10^{-16}$	0.005176	$< 2.2 \times 10^{-16}$	$< 2.2 \times 10^{-16}$	$< 2.2 \times 10^{-16}$	$< 2.2 \times 10^{-16}$	$< 2.2 \times 10^{-16}$	$< 2.2 \times 10^{-16}$	$< 2.2 \times 10^{-16}$	$< 2.2 \times 10^{-16}$	$< 2.2 \times 10^{-16}$	$< 2.2 \times 10^{-16}$	$< 2.2 \times 10^{-16}$	$< 2.2 \times 10^{-16}$	$< 2.2 \times 10^{-16}$	$< 2.2 \times 10^{-16}$	0.5124
	BCT	$< 2.2 \times 10^{-16}$	1	$< 2.2 \times 10^{-16}$	$< 2.2 \times 10^{-16}$	$< 2.2 \times 10^{-16}$	$< 2.2 \times 10^{-16}$	$< 2.2 \times 10^{-16}$	$< 2.2 \times 10^{-16}$	$< 2.2 \times 10^{-16}$	$< 2.2 \times 10^{-16}$	$< 2.2 \times 10^{-16}$	$< 2.2 \times 10^{-16}$	$< 2.2 \times 10^{-16}$	$< 2.2 \times 10^{-16}$	$< 2.2 \times 10^{-16}$	$< 2.2 \times 10^{-16}$	$< 2.2 \times 10^{-16}$	$< 2.2 \times 10^{-16}$	$< 2.2 \times 10^{-16}$	$< 2.2 \times 10^{-16}$	$< 2.2 \times 10^{-16}$
	CO2	$< 2.2 \times 10^{-16}$	$< 2.2 \times 10^{-16}$	1	$< 2.2 \times 10^{-16}$	$< 2.2 \times 10^{-16}$	$< 2.2 \times 10^{-16}$	$< 2.2 \times 10^{-16}$	$< 2.2 \times 10^{-16}$	$< 2.2 \times 10^{-16}$	$< 2.2 \times 10^{-16}$	$< 2.2 \times 10^{-16}$	$< 2.2 \times 10^{-16}$	$< 2.2 \times 10^{-16}$	$< 2.2 \times 10^{-16}$	$< 2.2 \times 10^{-16}$	$< 2.2 \times 10^{-16}$	$< 2.2 \times 10^{-16}$	$< 2.2 \times 10^{-16}$	$< 2.2 \times 10^{-16}$	$< 2.2 \times 10^{-16}$	$< 2.2 \times 10^{-16}$
**P236H**	Apo	$< 2.2 \times 10^{-16}$	$< 2.2 \times 10^{-16}$	$< 2.2 \times 10^{-16}$	$< 2.2 \times 10^{-16}$	$< 2.2 \times 10^{-16}$	$< 2.2 \times 10^{-16}$	$< 2.2 \times 10^{-16}$	$< 2.2 \times 10^{-16}$	$< 2.2 \times 10^{-16}$	$< 2.2 \times 10^{-16}$	$< 2.2 \times 10^{-16}$	$< 2.2 \times 10^{-16}$	1	$< 2.2 \times 10^{-16}$	$< 2.2 \times 10^{-16}$	$< 2.2 \times 10^{-16}$	$< 2.2 \times 10^{-16}$	$< 2.2 \times 10^{-16}$	$< 2.2 \times 10^{-16}$	$< 2.2 \times 10^{-16}$	$< 2.2 \times 10^{-16}$
	BCT	$< 2.2 \times 10^{-16}$	$< 2.2 \times 10^{-16}$	$< 2.2 \times 10^{-16}$	$< 2.2 \times 10^{-16}$	$< 2.2 \times 10^{-16}$	$< 2.2 \times 10^{-16}$	$< 2.2 \times 10^{-16}$	$< 2.2 \times 10^{-16}$	$< 2.2 \times 10^{-16}$	$< 2.2 \times 10^{-16}$	$< 2.2 \times 10^{-16}$	$< 2.2 \times 10^{-16}$	$< 2.2 \times 10^{-16}$	1	$< 2.2 \times 10^{-16}$	$< 2.2 \times 10^{-16}$	$< 2.2 \times 10^{-16}$	$< 2.2 \times 10^{-16}$	$< 2.2 \times 10^{-16}$	$< 2.2 \times 10^{-16}$	$< 2.2 \times 10^{-16}$
	CO2	$< 2.2 \times 10^{-16}$	$< 2.2 \times 10^{-16}$	$< 2.2 \times 10^{-16}$	$< 2.2 \times 10^{-16}$	$< 2.2 \times 10^{-16}$	$< 2.2 \times 10^{-16}$	$< 2.2 \times 10^{-16}$	$< 2.2 \times 10^{-16}$	$< 2.2 \times 10^{-16}$	$< 2.2 \times 10^{-16}$	$< 2.2 \times 10^{-16}$	$< 2.2 \times 10^{-16}$	$< 2.2 \times 10^{-16}$	$< 2.2 \times 10^{-16}$	1	$< 2.2 \times 10^{-16}$	$< 2.2 \times 10^{-16}$	$< 2.2 \times 10^{-16}$	$< 2.2 \times 10^{-16}$	$< 2.2 \times 10^{-16}$	$< 2.2 \times 10^{-16}$
**P236R**	Apo	$< 2.2 \times 10^{-16}$	$< 2.2 \times 10^{-16}$	$< 2.2 \times 10^{-16}$	$< 2.2 \times 10^{-16}$	$< 2.2 \times 10^{-16}$	$< 2.2 \times 10^{-16}$	$< 2.2 \times 10^{-16}$	$< 2.2 \times 10^{-16}$	$< 2.2 \times 10^{-16}$	$< 2.2 \times 10^{-16}$	$< 2.2 \times 10^{-16}$	$< 2.2 \times 10^{-16}$	$< 2.2 \times 10^{-16}$	$< 2.2 \times 10^{-16}$	$< 2.2 \times 10^{-16}$	1	0.02151	$< 2.2 \times 10^{-16}$	$< 2.2 \times 10^{-16}$	$< 2.2 \times 10^{-16}$	$< 2.2 \times 10^{-16}$
	BCT	$< 2.2 \times 10^{-16}$	$< 2.2 \times 10^{-16}$	$< 2.2 \times 10^{-16}$	$< 2.2 \times 10^{-16}$	$< 2.2 \times 10^{-16}$	$< 2.2 \times 10^{-16}$	$< 2.2 \times 10^{-16}$	$< 2.2 \times 10^{-16}$	$< 2.2 \times 10^{-16}$	$< 2.2 \times 10^{-16}$	$< 2.2 \times 10^{-16}$	$< 2.2 \times 10^{-16}$	$< 2.2 \times 10^{-16}$	$< 2.2 \times 10^{-16}$	$< 2.2 \times 10^{-16}$	0.02151	1	$< 2.2 \times 10^{-16}$	$< 2.2 \times 10^{-16}$	$< 2.2 \times 10^{-16}$	$< 2.2 \times 10^{-16}$
	CO2	$< 2.2 \times 10^{-16}$	$< 2.2 \times 10^{-16}$	$< 2.2 \times 10^{-16}$	$< 2.2 \times 10^{-16}$	$< 2.2 \times 10^{-16}$	$< 2.2 \times 10^{-16}$	$< 2.2 \times 10^{-16}$	$< 2.2 \times 10^{-16}$	$< 2.2 \times 10^{-16}$	$< 2.2 \times 10^{-16}$	$< 2.2 \times 10^{-16}$	$< 2.2 \times 10^{-16}$	$< 2.2 \times 10^{-16}$	$< 2.2 \times 10^{-16}$	$< 2.2 \times 10^{-16}$	$< 2.2 \times 10^{-16}$	$< 2.2 \times 10^{-16}$	1	$< 2.2 \times 10^{-16}$	$< 2.2 \times 10^{-16}$	$< 2.2 \times 10^{-16}$
**N252D**	Apo	$< 2.2 \times 10^{-16}$	$< 2.2 \times 10^{-16}$	$< 2.2 \times 10^{-16}$	$< 2.2 \times 10^{-16}$	$< 2.2 \times 10^{-16}$	$< 2.2 \times 10^{-16}$	$< 2.2 \times 10^{-16}$	$< 2.2 \times 10^{-16}$	$< 2.2 \times 10^{-16}$	1	$< 2.2 \times 10^{-16}$	2.373×10^{-10}	$< 2.2 \times 10^{-16}$	$< 2.2 \times 10^{-16}$	$< 2.2 \times 10^{-16}$	$< 2.2 \times 10^{-16}$	$< 2.2 \times 10^{-16}$	$< 2.2 \times 10^{-16}$	$< 2.2 \times 10^{-16}$	$< 2.2 \times 10^{-16}$	$< 2.2 \times 10^{-16}$
	BCT	$< 2.2 \times 10^{-16}$	$< 2.2 \times 10^{-16}$	$< 2.2 \times 10^{-16}$	$< 2.2 \times 10^{-16}$	$< 2.2 \times 10^{-16}$	$< 2.2 \times 10^{-16}$	$< 2.2 \times 10^{-16}$	$< 2.2 \times 10^{-16}$	$< 2.2 \times 10^{-16}$	$< 2.2 \times 10^{-16}$	1	$< 2.2 \times 10^{-16}$	$< 2.2 \times 10^{-16}$	$< 2.2 \times 10^{-16}$	$< 2.2 \times 10^{-16}$	$< 2.2 \times 10^{-16}$	$< 2.2 \times 10^{-16}$	$< 2.2 \times 10^{-16}$	$< 2.2 \times 10^{-16}$	$< 2.2 \times 10^{-16}$	$< 2.2 \times 10^{-16}$
	CO2	$< 2.2 \times 10^{-16}$	$< 2.2 \times 10^{-16}$	$< 2.2 \times 10^{-16}$	$< 2.2 \times 10^{-16}$	$< 2.2 \times 10^{-16}$	$< 2.2 \times 10^{-16}$	$< 2.2 \times 10^{-16}$	$< 2.2 \times 10^{-16}$	$< 2.2 \times 10^{-16}$	2.373×10^{-10}	$< 2.2 \times 10^{-16}$	1	$< 2.2 \times 10^{-16}$	$< 2.2 \times 10^{-16}$	$< 2.2 \times 10^{-16}$	$< 2.2 \times 10^{-16}$	$< 2.2 \times 10^{-16}$	$< 2.2 \times 10^{-16}$	$< 2.2 \times 10^{-16}$	$< 2.2 \times 10^{-16}$	$< 2.2 \times 10^{-16}$
**WT**	Apo	$< 2.2 \times 10^{-16}$	$< 2.2 \times 10^{-16}$	$< 2.2 \times 10^{-16}$	$< 2.2 \times 10^{-16}$	1.731×10^{-15}	$< 2.2 \times 10^{-16}$	$< 2.2 \times 10^{-16}$	$< 2.2 \times 10^{-16}$	$< 2.2 \times 10^{-16}$	$< 2.2 \times 10^{-16}$	$< 2.2 \times 10^{-16}$	$< 2.2 \times 10^{-16}$	$< 2.2 \times 10^{-16}$	$< 2.2 \times 10^{-16}$	$< 2.2 \times 10^{-16}$	$< 2.2 \times 10^{-16}$	$< 2.2 \times 10^{-16}$	$< 2.2 \times 10^{-16}$	1	$< 2.2 \times 10^{-16}$	6.227×10^{-15}
	BCT	$< 2.2 \times 10^{-16}$	$< 2.2 \times 10^{-16}$	$< 2.2 \times 10^{-16}$	$< 2.2 \times 10^{-16}$	$< 2.2 \times 10^{-16}$	$< 2.2 \times 10^{-16}$	$< 2.2 \times 10^{-16}$	$< 2.2 \times 10^{-16}$	$< 2.2 \times 10^{-16}$	$< 2.2 \times 10^{-16}$	$< 2.2 \times 10^{-16}$	$< 2.2 \times 10^{-16}$	$< 2.2 \times 10^{-16}$	$< 2.2 \times 10^{-16}$	$< 2.2 \times 10^{-16}$	$< 2.2 \times 10^{-16}$	$< 2.2 \times 10^{-16}$	$< 2.2 \times 10^{-16}$	$< 2.2 \times 10^{-16}$	1	$< 2.2 \times 10^{-16}$
	CO2	0.5124	$< 2.2 \times 10^{-16}$	$< 2.2 \times 10^{-16}$	$< 2.2 \times 10^{-16}$	$< 2.2 \times 10^{-16}$	5.517×10^{-07}	$< 2.2 \times 10^{-16}$	$< 2.2 \times 10^{-16}$	$< 2.2 \times 10^{-16}$	$< 2.2 \times 10^{-16}$	$< 2.2 \times 10^{-16}$	$< 2.2 \times 10^{-16}$	$< 2.2 \times 10^{-16}$	$< 2.2 \times 10^{-16}$	$< 2.2 \times 10^{-16}$	$< 2.2 \times 10^{-16}$	$< 2.2 \times 10^{-16}$	$< 2.2 \times 10^{-16}$	6.227×10^{-15}	$< 2.2 \times 10^{-16}$	1
**Radius of gyration (Rg)**																						
		H107Y			K18E			K18Q			N252D			P236H			P236R			WT		
		Apo	BCT	CO2	Apo	BCT	CO2	Apo	BCT	CO2	Apo	BCT	CO2	Apo	BCT	CO2	Apo	BCT	CO2	Apo	BCT	CO2
**K18E**	Apo	$< 2.2 \times 10^{-16}$	$< 2.2 \times 10^{-16}$	$< 2.2 \times 10^{-16}$	1	$< 2.2 \times 10^{-16}$	$< 2.2 \times 10^{-16}$	$< 2.2 \times 10^{-16}$	$< 2.2 \times 10^{-16}$	$< 2.2 \times 10^{-16}$	$< 2.2 \times 10^{-16}$	$< 2.2 \times 10^{-16}$	$< 2.2 \times 10^{-16}$	$< 2.2 \times 10^{-16}$	$< 2.2 \times 10^{-16}$	$< 2.2 \times 10^{-16}$	$< 2.2 \times 10^{-16}$	$< 2.2 \times 10^{-16}$	$< 2.2 \times 10^{-16}$	$< 2.2 \times 10^{-16}$	$< 2.2 \times 10^{-16}$	$< 2.2 \times 10^{-16}$
	BCT	$< 2.2 \times 10^{-16}$	$< 2.2 \times 10^{-16}$	$< 2.2 \times 10^{-16}$	$< 2.2 \times 10^{-16}$	1	$< 2.2 \times 10^{-16}$	$< 2.2 \times 10^{-16}$	$< 2.2 \times 10^{-16}$	$< 2.2 \times 10^{-16}$	$< 2.2 \times 10^{-16}$	$< 2.2 \times 10^{-16}$	$< 2.2 \times 10^{-16}$	$< 2.2 \times 10^{-16}$	$< 2.2 \times 10^{-16}$	2.325×10^{-09}	$< 2.2 \times 10^{-16}$	$< 2.2 \times 10^{-16}$	4.735×10^{-05}	$< 2.2 \times 10^{-16}$	$< 2.2 \times 10^{-16}$	$< 2.2 \times 10^{-16}$
	CO2	$< 2.2 \times 10^{-16}$	$< 2.2 \times 10^{-16}$	$< 2.2 \times 10^{-16}$	$< 2.2 \times 10^{-16}$	$< 2.2 \times 10^{-16}$	1	8.154×10^{-12}	$< 2.2 \times 10^{-16}$	$< 2.2 \times 10^{-16}$	$< 2.2 \times 10^{-16}$	$< 2.2 \times 10^{-16}$	3.049×10^{-10}	$< 2.2 \times 10^{-16}$	0.2254	$< 2.2 \times 10^{-16}$	$< 2.2 \times 10^{-16}$	4.226×10^{-09}	$< 2.2 \times 10^{-16}$	0.06527	3.08×10^{-14}	6.491×10^{-07}
**K18Q**	Apo	$< 2.2 \times 10^{-16}$	$< 2.2 \times 10^{-16}$	$< 2.2 \times 10^{-16}$	$< 2.2 \times 10^{-16}$	$< 2.2 \times 10^{-16}$	8.154×10^{-12}	1	$< 2.2 \times 10^{-16}$	$< 2.2 \times 10^{-16}$	$< 2.2 \times 10^{-16}$	$< 2.2 \times 10^{-16}$	0.5342	$< 2.2 \times 10^{-16}$	$< 2.2 \times 10^{-16}$	$< 2.2 \times 10^{-16}$	$< 2.2 \times 10^{-16}$	0.3209	$< 2.2 \times 10^{-16}$	1.342×10^{-07}	$< 2.2 \times 10^{-16}$	0.04238
	BCT	$< 2.2 \times 10^{-16}$	$< 2.2 \times 10^{-16}$	$< 2.2 \times 10^{-16}$	$< 2.2 \times 10^{-16}$	$< 2.2 \times 10^{-16}$	$< 2.2 \times 10^{-16}$	$< 2.2 \times 10^{-16}$	1	$< 2.2 \times 10^{-16}$	$< 2.2 \times 10^{-16}$	$< 2.2 \times 10^{-16}$	$< 2.2 \times 10^{-16}$	$< 2.2 \times 10^{-16}$	$< 2.2 \times 10^{-16}$	$< 2.2 \times 10^{-16}$	$< 2.2 \times 10^{-16}$	$< 2.2 \times 10^{-16}$	$< 2.2 \times 10^{-16}$	$< 2.2 \times 10^{-16}$	$< 2.2 \times 10^{-16}$	$< 2.2 \times 10^{-16}$
	CO2	$< 2.2 \times 10^{-16}$	$< 2.2 \times 10^{-16}$	$< 2.2 \times 10^{-16}$	$< 2.2 \times 10^{-16}$	$< 2.2 \times 10^{-16}$	$< 2.2 \times 10^{-16}$	$< 2.2 \times 10^{-16}$	$< 2.2 \times 10^{-16}$	1	$< 2.2 \times 10^{-16}$	$< 2.2 \times 10^{-16}$	$< 2.2 \times 10^{-16}$	$< 2.2 \times 10^{-16}$	$< 2.2 \times 10^{-16}$	$< 2.2 \times 10^{-16}$	$< 2.2 \times 10^{-16}$	$< 2.2 \times 10^{-16}$	$< 2.2 \times 10^{-16}$	$< 2.2 \times 10^{-16}$	$< 2.2 \times 10^{-16}$	$< 2.2 \times 10^{-16}$
**H107Y**	Apo	1	$< 2.2 \times 10^{-16}$	$< 2.2 \times 10^{-16}$	$< 2.2 \times 10^{-16}$	$< 2.2 \times 10^{-16}$	$< 2.2 \times 10^{-16}$	$< 2.2 \times 10^{-16}$	$< 2.2 \times 10^{-16}$	$< 2.2 \times 10^{-16}$	$< 2.2 \times 10^{-16}$	$< 2.2 \times 10^{-16}$	$< 2.2 \times 10^{-16}$	$< 2.2 \times 10^{-16}$	$< 2.2 \times 10^{-16}$	$< 2.2 \times 10^{-16}$	$< 2.2 \times 10^{-16}$	$< 2.2 \times 10^{-16}$	$< 2.2 \times 10^{-16}$	$< 2.2 \times 10^{-16}$	$< 2.2 \times 10^{-16}$	$< 2.2 \times 10^{-16}$
	BCT	$< 2.2 \times 10^{-16}$	1	$< 2.2 \times 10^{-16}$	$< 2.2 \times 10^{-16}$	$< 2.2 \times 10^{-16}$	$< 2.2 \times 10^{-16}$	$< 2.2 \times 10^{-16}$	$< 2.2 \times 10^{-16}$	$< 2.2 \times 10^{-16}$	$< 2.2 \times 10^{-16}$	$< 2.2 \times 10^{-16}$	$< 2.2 \times 10^{-16}$	$< 2.2 \times 10^{-16}$	$< 2.2 \times 10^{-16}$	$< 2.2 \times 10^{-16}$	$< 2.2 \times 10^{-16}$	$< 2.2 \times 10^{-16}$	$< 2.2 \times 10^{-16}$	$< 2.2 \times 10^{-16}$	$< 2.2 \times 10^{-16}$	$< 2.2 \times 10^{-16}$
	CO2	$< 2.2 \times 10^{-16}$	$< 2.2 \times 10^{-16}$	1	$< 2.2 \times 10^{-16}$	$< 2.2 \times 10^{-16}$	$< 2.2 \times 10^{-16}$	$< 2.2 \times 10^{-16}$	$< 2.2 \times 10^{-16}$	$< 2.2 \times 10^{-16}$	$< 2.2 \times 10^{-16}$	$< 2.2 \times 10^{-16}$	$< 2.2 \times 10^{-16}$	$< 2.2 \times 10^{-16}$	$< 2.2 \times 10^{-16}$	$< 2.2 \times 10^{-16}$	$< 2.2 \times 10^{-16}$	$< 2.2 \times 10^{-16}$	$< 2.2 \times 10^{-16}$	$< 2.2 \times 10^{-16}$	$< 2.2 \times 10^{-16}$	$< 2.2 \times 10^{-16}$
**P236H**	Apo	$< 2.2 \times 10^{-16}$	$< 2.2 \times 10^{-16}$	$< 2.2 \times 10^{-16}$	$< 2.2 \times 10^{-16}$	$< 2.2 \times 10^{-16}$	$< 2.2 \times 10^{-16}$	$< 2.2 \times 10^{-16}$	$< 2.2 \times 10^{-16}$	$< 2.2 \times 10^{-16}$	$< 2.2 \times 10^{-16}$	1.765×10^{-13}	$< 2.2 \times 10^{-16}$	1	$< 2.2 \times 10^{-16}$	0.0001396	$< 2.2 \times 10^{-16}$	$< 2.2 \times 10^{-16}$	$< 2.2 \times 10^{-16}$	$< 2.2 \times 10^{-16}$	$< 2.2 \times 10^{-16}$	$< 2.2 \times 10^{-16}$
	BCT	$< 2.2 \times 10^{-16}$	$< 2.2 \times 10^{-16}$	$< 2.2 \times 10^{-16}$	$< 2.2 \times 10^{-16}$	$< 2.2 \times 10^{-16}$	0.2254	$< 2.2 \times 10^{-16}$	$< 2.2 \times 10^{-16}$	$< 2.2 \times 10^{-16}$	$< 2.2 \times 10^{-16}$	$< 2.2 \times 10^{-16}$	5.635×10^{-15}	$< 2.2 \times 10^{-16}$	1	$< 2.2 \times 10^{-16}$	$< 2.2 \times 10^{-16}$	3.696×10^{-13}	$< 2.2 \times 10^{-16}$	0.0009534	2.425×10^{-10}	1.059×10^{-10}
	CO2	$< 2.2 \times 10^{-16}$	$< 2.2 \times 10^{-16}$	$< 2.2 \times 10^{-16}$	$< 2.2 \times 10^{-16}$	2.325×10^{-09}	$< 2.2 \times 10^{-16}$	$< 2.2 \times 10^{-16}$	$< 2.2 \times 10^{-16}$	$< 2.2 \times 10^{-16}$	$< 2.2 \times 10^{-16}$	$< 2.2 \times 10^{-16}$	$< 2.2 \times 10^{-16}$	0.0001396	$< 2.2 \times 10^{-16}$	1	$< 2.2 \times 10^{-16}$	$< 2.2 \times 10^{-16}$	$< 2.2 \times 10^{-16}$	$< 2.2 \times 10^{-16}$	$< 2.2 \times 10^{-16}$	$< 2.2 \times 10^{-16}$
**P236R**	Apo	$< 2.2 \times 10^{-16}$	$< 2.2 \times 10^{-16}$	$< 2.2 \times 10^{-16}$	$< 2.2 \times 10^{-16}$	$< 2.2 \times 10^{-16}$	$< 2.2 \times 10^{-16}$	$< 2.2 \times 10^{-16}$	$< 2.2 \times 10^{-16}$	$< 2.2 \times 10^{-16}$	$< 2.2 \times 10^{-16}$	$< 2.2 \times 10^{-16}$	$< 2.2 \times 10^{-16}$	$< 2.2 \times 10^{-16}$	$< 2.2 \times 10^{-16}$	$< 2.2 \times 10^{-16}$	1	$< 2.2 \times 10^{-16}$	2.607×10^{-13}	$< 2.2 \times 10^{-16}$	$< 2.2 \times 10^{-16}$	$< 2.2 \times 10^{-16}$
	BCT	$< 2.2 \times 10^{-16}$	$< 2.2 \times 10^{-16}$	$< 2.2 \times 10^{-16}$	$< 2.2 \times 10^{-16}$	$< 2.2 \times 10^{-16}$	4.226×10^{-09}	0.3209	$< 2.2 \times 10^{-16}$	$< 2.2 \times 10^{-16}$	$< 2.2 \times 10^{-16}$	$< 2.2 \times 10^{-16}$	0.6927	$< 2.2 \times 10^{-16}$	3.696×10^{-13}	$< 2.2 \times 10^{-16}$	$< 2.2 \times 10^{-16}$	1	$< 2.2 \times 10^{-16}$	2.766×10^{-05}	$< 2.2 \times 10^{-16}$	0.3288
	CO2	$< 2.2 \times 10^{-16}$	$< 2.2 \times 10^{-16}$	$< 2.2 \times 10^{-16}$	$< 2.2 \times 10^{-16}$	4.735×10^{-05}	$< 2.2 \times 10^{-16}$	$< 2.2 \times 10^{-16}$	$< 2.2 \times 10^{-16}$	$< 2.2 \times 10^{-16}$	$< 2.2 \times 10^{-16}$	$< 2.2 \times 10^{-16}$	$< 2.2 \times 10^{-16}$	$< 2.2 \times 10^{-16}$	$< 2.2 \times 10^{-16}$	$< 2.2 \times 10^{-16}$	2.607×10^{-13}	$< 2.2 \times 10^{-16}$	1	$< 2.2 \times 10^{-16}$	$< 2.2 \times 10^{-16}$	$< 2.2 \times 10^{-16}$
**N252D**	Apo	$< 2.2 \times 10^{-16}$	$< 2.2 \times 10^{-16}$	$< 2.2 \times 10^{-16}$	$< 2.2 \times 10^{-16}$	$< 2.2 \times 10^{-16}$	$< 2.2 \times 10^{-16}$	$< 2.2 \times 10^{-16}$	$< 2.2 \times 10^{-16}$	$< 2.2 \times 10^{-16}$	1	$< 2.2 \times 10^{-16}$	$< 2.2 \times 10^{-16}$	$< 2.2 \times 10^{-16}$	$< 2.2 \times 10^{-16}$	$< 2.2 \times 10^{-16}$	$< 2.2 \times 10^{-16}$	$< 2.2 \times 10^{-16}$	$< 2.2 \times 10^{-16}$	$< 2.2 \times 10^{-16}$	$< 2.2 \times 10^{-16}$	$< 2.2 \times 10^{-16}$
	BCT	$< 2.2 \times 10^{-16}$	$< 2.2 \times 10^{-16}$	$< 2.2 \times 10^{-16}$	$< 2.2 \times 10^{-16}$	$< 2.2 \times 10^{-16}$	$< 2.2 \times 10^{-16}$	$< 2.2 \times 10^{-16}$	$< 2.2 \times 10^{-16}$	$< 2.2 \times 10^{-16}$	$< 2.2 \times 10^{-16}$	1	$< 2.2 \times 10^{-16}$	1.765×10^{-13}	$< 2.2 \times 10^{-16}$	$< 2.2 \times 10^{-16}$	$< 2.2 \times 10^{-16}$	$< 2.2 \times 10^{-16}$	$< 2.2 \times 10^{-16}$	$< 2.2 \times 10^{-16}$	$< 2.2 \times 10^{-16}$	$< 2.2 \times 10^{-16}$
	CO2	$< 2.2 \times 10^{-16}$	$< 2.2 \times 10^{-16}$	$< 2.2 \times 10^{-16}$	$< 2.2 \times 10^{-16}$	$< 2.2 \times 10^{-16}$	3.049×10^{-10}	0.5342	$< 2.2 \times 10^{-16}$	$< 2.2 \times 10^{-16}$	$< 2.2 \times 10^{-16}$	$< 2.2 \times 10^{-16}$	1	$< 2.2 \times 10^{-16}$	5.635×10^{-15}	$< 2.2 \times 10^{-16}$	$< 2.2 \times 10^{-16}$	0.6927	$< 2.2 \times 10^{-16}$	2.68×10^{-06}	$< 2.2 \times 10^{-16}$	0.1479
**WT**	Apo	$< 2.2 \times 10^{-16}$	$< 2.2 \times 10^{-16}$	$< 2.2 \times 10^{-16}$	$< 2.2 \times 10^{-16}$	$< 2.2 \times 10^{-16}$	0.06527	1.342×10^{-07}	$< 2.2 \times 10^{-16}$	$< 2.2 \times 10^{-16}$	$< 2.2 \times 10^{-16}$	$< 2.2 \times 10^{-16}$	2.68×10^{-06}	$< 2.2 \times 10^{-16}$	0.0009534	$< 2.2 \times 10^{-16}$	$< 2.2 \times 10^{-16}$	2.766×10^{-05}	$< 2.2 \times 10^{-16}$	1	$< 2.2 \times 10^{-16}$	0.001236
	BCT	$< 2.2 \times 10^{-16}$	$< 2.2 \times 10^{-16}$	$< 2.2 \times 10^{-16}$	$< 2.2 \times 10^{-16}$	$< 2.2 \times 10^{-16}$	3.08×10^{-14}	$< 2.2 \times 10^{-16}$	$< 2.2 \times 10^{-16}$	$< 2.2 \times 10^{-16}$	$< 2.2 \times 10^{-16}$	$< 2.2 \times 10^{-16}$	$< 2.2 \times 10^{-16}$	$< 2.2 \times 10^{-16}$	2.425×10^{-10}	$< 2.2 \times 10^{-16}$	$< 2.2 \times 10^{-16}$	$< 2.2 \times 10^{-16}$	$< 2.2 \times 10^{-16}$	$< 2.2 \times 10^{-16}$	1	$< 2.2 \times 10^{-16}$
	CO2	$< 2.2 \times 10^{-16}$	$< 2.2 \times 10^{-16}$	$< 2.2 \times 10^{-16}$	$< 2.2 \times 10^{-16}$	$< 2.2 \times 10^{-16}$	6.491×10^{-07}	0.04238	$< 2.2 \times 10^{-16}$	$< 2.2 \times 10^{-16}$	$< 2.2 \times 10^{-16}$	$< 2.2 \times 10^{-16}$	0.1479	$< 2.2 \times 10^{-16}$	1.059×10^{-10}	$< 2.2 \times 10^{-16}$	$< 2.2 \times 10^{-16}$	0.3288	$< 2.2 \times 10^{-16}$	0.001236	$< 2.2 \times 10^{-16}$	1

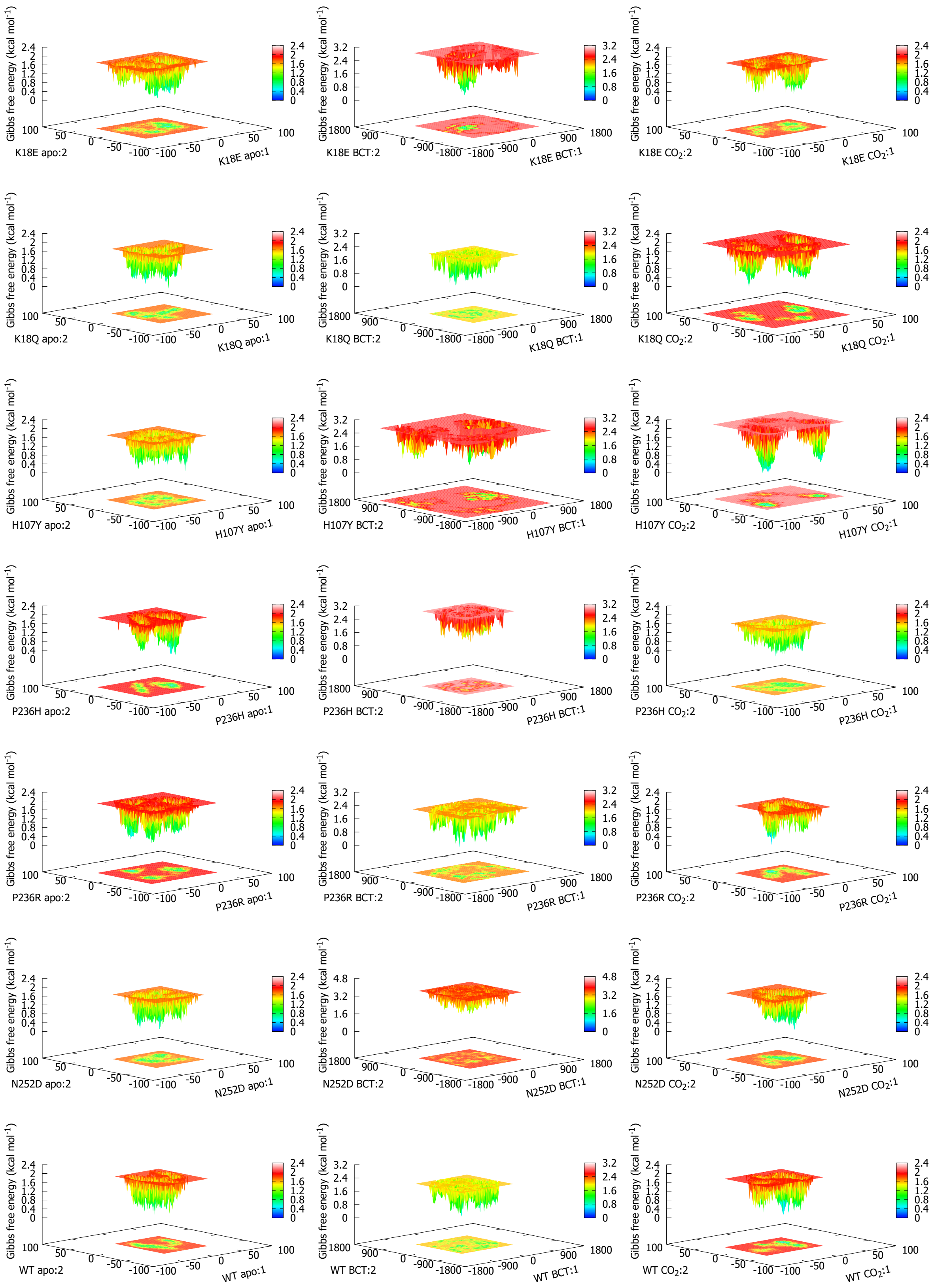

Figure S3. 3-dimensional (3D) plot of PC1 vs PC2 of the WT and variants proteins as a function of free energy. 2D PCA is projected onto the x-axis and *y*-axis. Free energy is represented in kcal mol^{-1}.

Table S5. Eigenvalue fraction of each principal component for the active site residues within the wild-type and variant proteins in the absence (apo) and presence (non apo) of CO_2.

Variant	Protein	PC1	PC2	PC3
**K18E**	Apo	0.5503	0.2500	0.1997
	BCT	0.4836	0.3165	0.1999
	CO_2	0.5772	0.2218	0.2010
**K18Q**	Apo	0.5260	0.2732	0.2009
	BCT	0.4497	0.3598	0.1905
	CO_2	0.6091	0.2647	0.1261
**H107Y**	Apo	0.4439	0.3322	0.2239
	BCT	0.5688	0.2764	0.1548
	CO_2	0.7201	0.1670	0.1129
**P236H**	Apo	0.5535	0.2628	0.1837
	BCT	0.5195	0.2741	0.2064
	CO_2	0.4846	0.2977	0.2177
**P236R**	Apo	0.4693	0.3107	0.2200
	BCT	0.4772	0.3415	0.1813
	CO_2	0.4218	0.3208	0.2574
**N252D**	Apo	0.4415	0.3358	0.2227
	BCT	0.4635	0.3607	0.1758
	CO_2	0.4348	0.3235	0.2417
**WT**	Apo	0.4673	0.3253	0.2074
	BCT	0.4256	0.3550	0.2194
	CO_2	0.5399	0.2589	0.2012

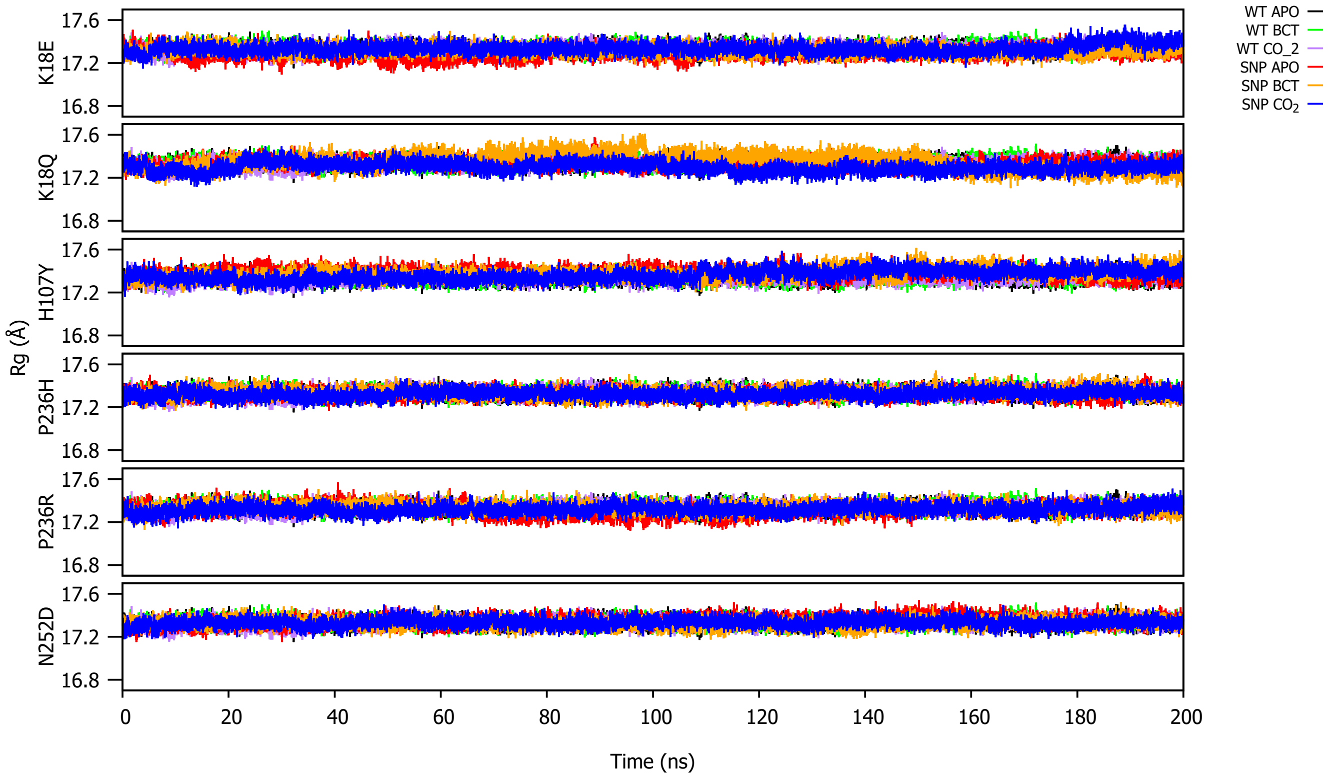

Figure S4. Rg comparison between the WT and variant proteins during MD.

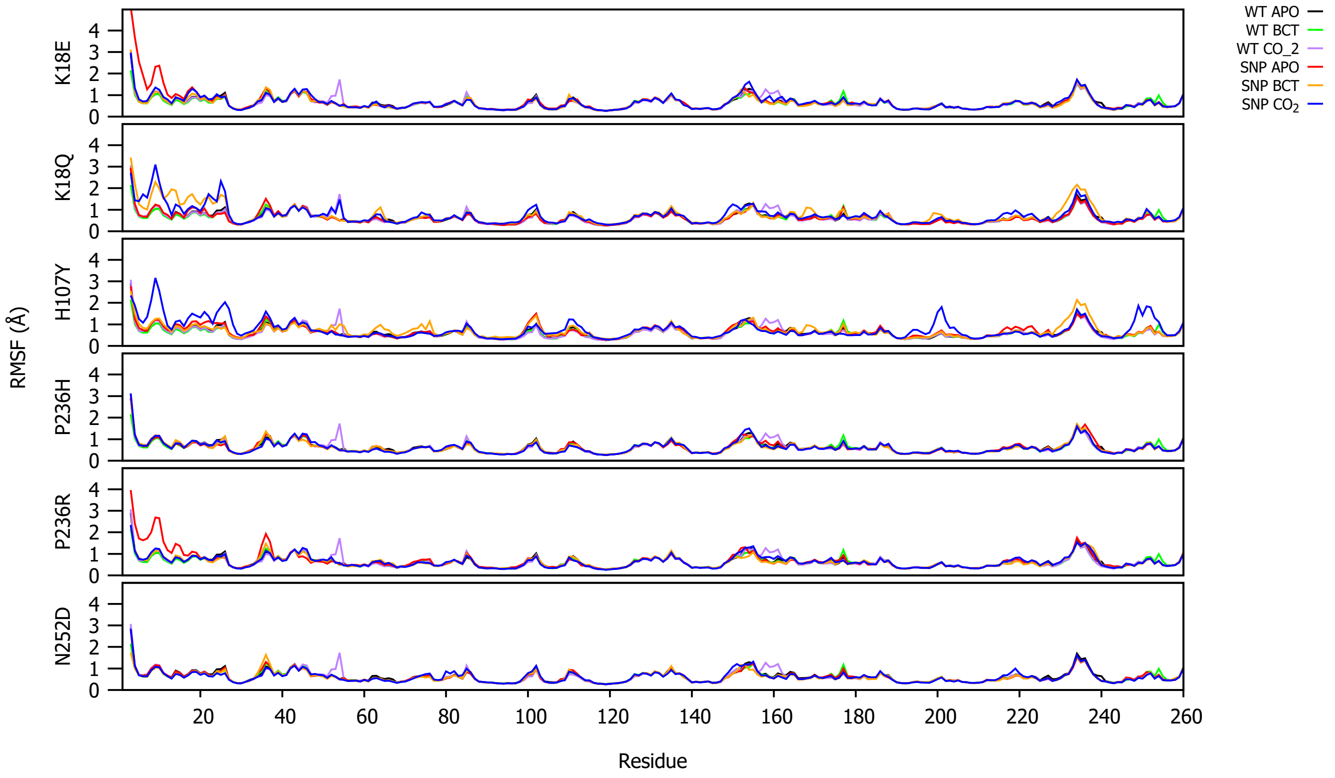

Figure S5. Residue RMSF comparison between the WT and variant proteins.

Table S6. Variant residues showing decreases and increases to Δ*L* during MD simulation. Residues from Table reftab:residues are underlined and highlighted in bold. SNV positions are underlined, italicised and highlighted in bold red.

Variant	Apo	BCT	CO_2
	**Δ*L* decrease (Residue accessibility increase)**		
**K18E**	His4 His10 **Trp16** Ile22 Gly170 Phe230 Asn231 Glu235 Pro236 Glu237 Glu238	Lys111 Gly155 Leu156	Ala54 Lys158 Val159 Val160 Asp161 Leu163 Asp164 Ser165 Ile166 Asn177 Phe178
**K18Q**	His3 Ile22 Ala152	His3 His4 **Trp5** Gly6 Gly8 Lys9 His10 Asn11 Lys24 Lys111	His3 His4 Lys9 Lys24 Lys158 Val159 Val160 Asp161 Leu163 Asp164 Ser165 Ile166
**H107Y**	Lys24	Val49 Ser50 Asp52 Gln53 Lys111 Leu184	Asp52 Gln53 Gly155 Lys158 Val159 Val160 Asp161 Val162 Leu163 Ser165 Ile166 Asp179 Pro180
**P236H**	Ile22 Asp101 Gly155 Thr199 His236 Glu237 Glu238	His3 Gly155 Leu156 Phe178 Asp179 Pro180 Arg181 Leu183 Pro201	His3 Ala54 Lys158 Val159 Val160 Asp161 Val162 Leu163 Asp164 Ser165 Ile166 Ala173 Phe175 Asn177 Glu237
**P236R**	Pro21 Ile22 Phe230 Asn231 Glu235 Arg236 Glu237 Glu238	Val49 Asp52 Gln53 Pro180	Asp52 Ile59 Lys158 Val159 Val160 Asp161 Val162 Leu163 Ile166 Pro180 Glu237
**N252D**	Leu163 Asp164	Lys111 Gly155 Leu156 Gln157 Leu183	Ala54 Lys158 Val159 Val160 Asp161 Val162 Leu163 Asp164 Ser165 Ile166 Phe175
	**Δ*L* increase (Residue accessibility decrease)**		
**K18E**	**Trp5** Lys111 Lys153 Leu188	Ala152 Lys153	Gly155 Leu156 Leu188
**K18Q**	Thr35 Lys111 Pro154 Gly155 Leu156 Lys158 Val159 Pro180 Gly182 Leu183 Glu220	Gly12 Pro13 Glu14 Asp19 Leu163 Glu233	Ile22 Ala54 Ala152
**H107Y**	**Ser29** Val31 Asp101 Lys111 Leu140 Ala152 Lys153 Val241	Val31 Ala54 Lys158	Gly25 Glu26 Arg27 Ala54 Lys111 Gln248 Lys251
**P236H**	His3 Thr35 Ala152 Lys158 Val159 Val160	His36 Thr37 Leu163 Asp164 Gly170	Leu156 **Phe225** Arg226
**P236R**	His3 Gly6 **Tyr7** Thr35 Gly98 Ser99 Leu100 Lys111 Asp242	Ala54 Leu163 Asp164 Gly170 **Thr199** Gly232 Gly234	Ala54 Leu156 Gly170
**N252D**	His3 Thr35 Gly63 Pro154 Gly155 Leu156 Gln157 Lys158 Pro180 Leu183	His3 Thr35 Ser43 Ala152 Lys153	Gly155 Leu156 Arg226

Table S7. Variant residues showing decreases and increases to ΔBC during MD simulation. Residues from Table S1 are underlined and highlighted in bold. SNV positions are underlined, italicised and highlighted in bold red.

Variant	Apo	BCT	CO_2
**ΔBC decrease (reduction in residue communication)**			
**K18E**	**Trp5** Gly6 Pro200 Met240 Asp242 **Asn243**	**His64** His107 Lys113 **Glu117** Ile145 Leu147 Lys153 **Thr199** Ile215 Val222	Asn61 Ala77 Leu90 Val162 Gly182 Leu183 Val217 **Phe225**
**K18Q**	Gly63 **His94** **Glu117** **His119** Leu147 Pro180 Leu183 Asp242 Trp244	**Trp5** Gly12 Pro13 **Trp16** **Phe66** **Asn67** His107 **Glu117** Thr168 Asp242 **Asn243** Pro246	**Trp5** Ala54 Leu60 Asn61 **His64** Phe70 Gly182 **Phe225** Asp242
**H107Y**	Val31 Gly63 **His94** **Glu117** **His119** Leu140 **Val142** Gly144	Val31 Ala54 **Phe66** ***Tyr107*** **Glu117** Gly144 **Thr199**	Ile33 Ala54 **His64** Glu69 **Glu117** Leu147 **Thr199** Val222 **Phe225**
**P236H**	Gly63 Val68 **Gln92** Val121 **Val142** Leu147 Val159 Asp242	**Phe66** **Phe70** **Gln92** **Phe95** **Glu117** Leu163	Asn61 Ala77 Leu90 Leu147 Gly182 Leu183 Val222 **Phe225**
**P236R**	Gly6 **His94** Ser105 **His119** **Val142** Asp242 **Asn243**	Tyr51 Ala54 **Asn67** **Phe95** **Glu117** Leu118 Leu147 **Thr199**	Ala54 Leu60 Asn61 Glu69 Gly182 Leu183 **Phe225**
**N252D**	Gly63 **His64** **His94** **His119** Leu156 Pro180 Leu183 **Thr199** **Phe225**	**His64** His107 **Glu117** Leu147 Lys153 **Thr199** Ile215	Leu60 Asn61 **Phe70** Ala77 Gly182 **Phe225**
**ΔBC increase (residue communication increase)**			
**K18E**	His4 **Trp16** **His96** **Thr199** Phe230	Ser105 Ala116 Leu118 Gly155 Leu156 Leu183 Val210 Val217	Ala54 Ala65 Val68 **Phe93** **Phe95** Ala116 Leu118 Val159 **Thr199**
**K18Q**	**Phe66** **His96** Lys113 Ala116 Ile145 Ile215 Val217	Gly8 Asn11 **His94** Ser105 Lys169 Phe230 Trp244 Gln248	**Tyr7** Gly8 Asn11 Ala65 **Phe66** Val68 **His96** Val160 Leu163 **Thr199** Phe230
**H107Y**	**Phe66** **Asn67** **Phe95** Glu106 Leu118 Val217	Thr55 Ala77 Leu90 **His94** Ser105 Val210	Ala65 **Phe66** **Phe93** **Phe95** **His96** Glu106 Ala116 Asp179
**P236H**	**His64** **Phe66** **His96** Ala116 Gly155 **Thr199** Val217	Gly63 **Phe93** **His94** **Trp97** Val134	Ala54 Ala65 **Phe66** **Phe93** **Phe95** Ala116 Leu163
**P236R**	**Tyr7** **Phe66** **Phe95** **His96** Glu106 **Thr199** Phe230 **Arg245**	Ala77 **His94** Ala116 **His119** Val217	Ala65 **Phe66** **His94** Ala116 Leu163 Phe175 Pro180
**N252D**	**Phe66** **Asn67** **Gln92** **Phe95** **His96** Ile215 **Asn243**	Ser105 Ala116 Gly155 Leu156 Leu183 Val217 **Asn243** Trp244	Tyr51 Ala54 Ala65 **Phe66** Val68 Ile91 **Gln92** **Phe93** **Val142** Val160 Leu163 Ile215

References (Table S1)

1. Lindskog, S. Structure and mechanism of carbonic anhydrase. *Pharmacology & therapeutics* **1997**, *74*, 1–20.
2. Eriksson, A.E.; Jones, T.A.; Liljas, A. Refined structure of human carbonic anhydrase II at 2.0 Å resolution. *Proteins: Structure, Function, and Bioinformatics* **1988**, *4*, 274–282.
3. Hunt, J.A.; Fierke, C.A. Selection of carbonic anhydrase variants displayed on phage aromatic residues in zinc binding site enhance metal affinity and equilibration kinetics. *Journal of Biological Chemistry* **1997**, *272*, 20364–20372.
4. Silverman, D.N.; McKenna, R. Solvent-mediated proton transfer in catalysis by carbonic anhydrase. *Accounts of chemical research* **2007**, *40*, 669–675.
5. Merz Jr, K.M. Carbon dioxide binding to human carbonic anhydrase II. *Journal of the American Chemical Society* **1991**, *113*, 406–411.
6. Liang, J.Y.; Lipscomb, W.N. Binding of substrate CO2 to the active site of human carbonic anhydrase II: a molecular dynamics study. *Proceedings of the National Academy of Sciences* **1990**, *87*, 3675–3679.
7. Domsic, J.F.; Avvaru, B.S.; Kim, C.U.; Gruner, S.M.; Agbandje-McKenna, M.; Silverman, D.N.; McKenna, R. Entrapment of carbon dioxide in the active site of carbonic anhydrase II. *Journal of Biological Chemistry* **2008**, *283*, 30766–30771.
8. Alexander, R.S.; Nair, S.K.; Christianson, D.W. Engineering the hydrophobic pocket of carbonic anhydrase II. *Biochemistry* **1991**, *30*, 11064–11072.
9. Mikulski, R.L.; Silverman, D.N. Proton transfer in catalysis and the role of proton shuttles in carbonic anhydrase. *Biochimica et Biophysica Acta (BBA)-Proteins and Proteomics* **2010**, *1804*, 422–426.
10. Mårtensson, L.G.; Jonsson, B.H.; Andersson, M.; Kihlgren, A.; Bergenhem, N.; Carlsson, U. Role of an evolutionarily invariant serine for the stability of human carbonic anhydrase II. *Biochimica et Biophysica Acta (BBA)-Protein Structure and Molecular Enzymology* **1992**, *1118*, 179–186.
11. Almstedt, K.; Lundqvist, M.; Carlsson, J.; Karlsson, M.; Persson, B.; Jonsson, B.H.; Carlsson, U.; Hammarström, P. Unfolding a folding disease: folding, misfolding and aggregation of the marble brain syndrome-associated mutant H107Y of human carbonic anhydrase II. *Journal of molecular biology* **2004**, *342*, 619–633.
12. Silverman, D.N.; Lindskog, S. The catalytic mechanism of carbonic anhydrase: implications of a rate-limiting protolysis of water. *Accounts of chemical research* **1988**, *21*, 30–36.
13. Kiefer, L.L.; Paterno, S.A.; Fierke, C.A. Hydrogen bond network in the metal binding site of carbonic anhydrase enhances zinc affinity and catalytic efficiency. *Journal of the american chemical society* **1995**, *117*, 6831–6837.
14. Tashian, R.E. Genetics of the mammalian carbonic anhydrases. In *Advances in genetics*; Elsevier, 1992; Vol. 30, pp. 321–356.
